# Mesocorticostriatal Reinforcement Learning of State Representation and Value with Implications for the Mechanisms of Schizophrenia

**DOI:** 10.1523/JNEUROSCI.1762-25.2026

**Published:** 2026-03-03

**Authors:** Kenji Morita, Arvind Kumar

**Affiliations:** ^1^Physical and Health Education, Graduate School of Education, The University of Tokyo, Tokyo 113-0033, Japan; ^2^International Research Center for Neurointelligence (WPI-IRCN), The University of Tokyo, Tokyo 113-0033, Japan; ^3^Division of Computational Science and Technology, School of Electrical Engineering and Computer Science, KTH Royal Institute of Technology, Stockholm SE-100 44, Sweden; ^4^Science for Life Laboratory, Solna 17121, Sweden

**Keywords:** dopamine, excitation/inhibition balance, feedback alignment, recurrent neural networks, reinforcement learning, schizophrenia

## Abstract

Mesocorticostriatal dopamine projections are crucial for value learning, motivational control, and cognitive functions. However, while dopamine's role in value learning as reward-prediction-error (RPE) has been much understood, precise roles in motivational control and cognitive functions remain more elusive. Computationally, this corresponds to that while the operation of mesostriatal dopamine could be minimally described by simple reinforcement learning (RL) models with one-dimensional reward/RPE and fixed state representation, (1) how reward-specific motivational control can be achieved through heterogeneous dopamine responses, and (2) how sophisticated cortical state representation can be formed through mesocortical dopamine, cannot be captured by such simple models. To address both of these at once, we combined recent models for each of them: the “Reward Bases (RB),” which achieved reward-specific motivational control through multidimensional RPE (but with fixed cortical representation), and the “online value-recurrent-neutral-network (OVRNN),” which achieved state representation learning through training of RNN by RPE (but of one-dimensional). We show the combined model can achieve both functions simultaneously via double “feedback alignments” of the cortical and striatal downstream connections to the mesocorticostriatal dopamine projections. Crucially, cortical inhibition-dominance is a key for successful learning. Excessive excitation leads to aberrant persistent activity, which disrupts the alignments and impairs reward-specific motivational control and credit assignment. This implies how negative and positive symptoms of schizophrenia could emerge from excitation/inhibition imbalance, and we show how our model could explain altered brain activations in patients. Our model thus provides an integrated computational account for dopamine's functions, with implications on how its dysfunctions link to schizophrenia.

## Significance Statement

Dopamine has been suggested to play crucial roles in value learning, motivational control, and cognitive functions, and they have been tried to be understood using the reinforcement learning (RL) framework. However, existing RL models have two limitations: reward identity/diversity is ignored, and state/action representation is handcrafted. Recent studies addressed either of them, but only separately. We combine these separate models and demonstrate that reward-specific value and state representation can be simultaneously learned through double operations of “feedback alignment,” a bioplausible alternative to the dominant machine-learning algorithm. Crucially, inhibition-dominance is a key for successful learning. Excessive excitation-induced persistent activity disturbs alignments and impairs motivational control and credit assignment, implying how excitation/inhibition imbalance could lead to negative and positive symptoms of schizophrenia.

## Introduction

**Figure 1.**
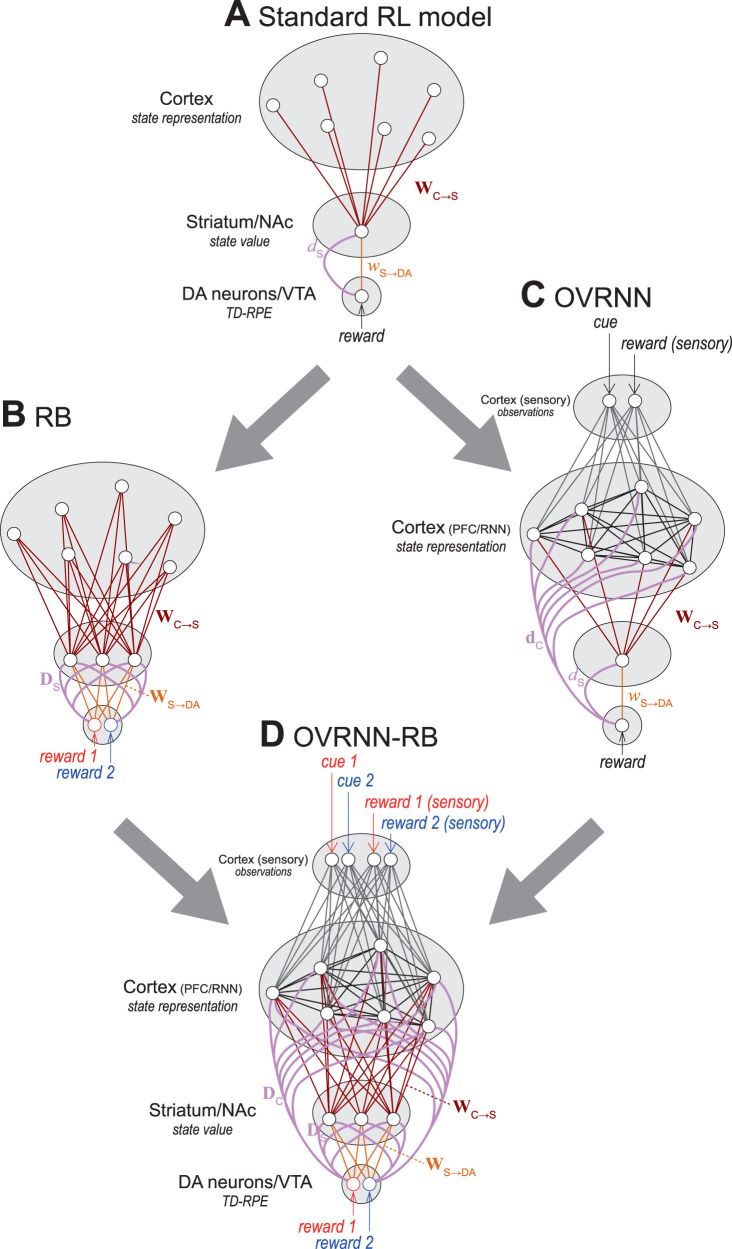
Development of reinforcement learning (RL)-based neural circuit models to understand the diverse functions of dopamine (DA). ***A***, Standard RL model, which learns the value of scalar (one-dimensional) reward with fixed state representation. ***B***, The reward bases (RB) model (Millidge et al., 2024), which incorporated heterogeneous DA neurons encoding multidimensional RPE and achieved reward-specific motivational control through the “alignment” of the striatum → DA connections (**W**_S → DA_) to the DA → striatum (i.e., mesostriatal) projections (**D**_S_). ***C***, The Online Value-RNN (OVRNN) model (Tsurumi et al., 2025), which incorporated training of cortical RNN by DA/RPE and achieved learning of task-appropriate state representation through the “alignment” of the cortex → DA connections (**W**_C → S_*w*_S → DA_) to the DA → cortex (i.e., mesocortical) projections (**d**_C_). ***D***, The model presented in this work (OVRNN-RB), which combines the RB and OVRNN models. We show that this model simultaneously achieves reward-specific value learning (motivational control) and learning of task-appropriate state representation through double alignments of the striatum → DA connections (**W**_S → DA_) to the DA → striatum projections (**D**_S_) and the cortex → DA connections (**W**_C → S_**W**_S → DA_) to the DA → cortex projections (**D**_C_).

Mesocorticostriatal dopamine (DA) has been suggested to be crucial for value learning, motivational control, and cognitive functions. Among them, the role of mesostriatal DA in value learning has largely been established under the reinforcement learning (RL) framework: DA encodes reward-prediction-error (RPE) and its modulation of corticostriatal plasticity implements RPE-dependent update of value prediction [Montague et al., 1996; Schultz et al., 1997; Reynolds et al., 2001; although further consideration continues (Hamid et al., 2016; Jeong et al., 2022; de Jong et al., 2024; Gershman et al., 2024; Kato and Morita, 2025; Qian et al., 2025); Fig. 1*A*].

Compared with this, DA's roles in motivational and cognitive controls remain more elusive. A key feature of motivational control is its specificity to reward identity—seeking food when hungry and drink when thirsty—and reward identity-dependence of DA signals has been demonstrated (Takahashi et al., 2017; Howard and Kahnt, 2018; Kahnt and Schoenbaum, 2025). On the contrary, conventional RL describes reward as one-dimensional scalar variable, ignoring reward identity/diversity. Regarding DA's cognitive functions, early studies focused on working memory (Cools and D'Esposito, 2011), but DA also relates to wider functions (Puig and Miller, 2012; Puig et al., 2014; Ott and Nieder, 2019), including context representation (D'Ardenne et al., 2012) and flexible behavior (apparently) based on the internal model of the environment (Langdon et al., 2018). Whether such DA's motivational and cognitive functions can also be understood in the RL framework has remained elusive.

In other words, while the operation of mesostriatal DA could be minimally described by simple RL models with one-dimensional reward/RPE and fixed state representation, it remains unclear: (1) how can reward-specific motivational control be achieved through heterogeneous DA responses? and (2) how can sophisticated cortical state representation be formed through mesocortical DA? Addressing these questions could potentially provide insights into psychiatric disorders which involve DA dysfunction such as schizophrenia.

Recently, advanced RL models have been developed to account for either the motivational or cognitive function of DA, i.e., to address either of the abovementioned two limitations of the standard RL models, although separately. To address the problem of reward-specific motivational function, the reward bases (RB) model (Millidge et al., 2024) was developed in reference to reward identity-dependence of DA signals (Takahashi et al., 2017; Howard and Kahnt, 2018; Kahnt and Schoenbaum, 2025) and previous models (Collins and Frank, 2014; Keramati and Gutkin, 2014; Möller and Bogacz, 2019; Wärnberg and Kumar, 2023; Fig. 1*B*). RB incorporates multidimensional RPE encoding by DA neuronal populations, which enables reward-specific motivational control. To explain sophisticated cortical state representation and other cognitive or model-based(-like) functions, models in which RPE trains cortical recurrent neural network (RNN) have been proposed (J. X. Wang et al., 2018; Hennig et al., 2023) and compared with experimental data (Hattori et al., 2023). Recently, we developed the online value-RNN (OVRNN; Tsurumi et al., 2025), which uses biologically plausible online local feedback instead of artificial learning rules used in the previous models including the original value-RNN (Hennig et al., 2023; Fig. 1*C*). OVRNN enables learning of task-appropriate state representation and value by training of cortical RNN and its readout (striatum) by RPE.

RB does not deal with cortical DA's functions, while OVRNN sticks to one-dimensional RPE, and so integration of these two models is desired. However, operation of RB is based on the “alignment” of the striatum-DA connections to the backward mesostriatal connections, whereas OVRNN is based on the “alignment” of the corticostriatal connections to the mesocortical connections, and whether both alignments can simultaneously occur is nontrivial. In the present study, we addressed this issue. We developed a model of the mesocorticostriatal system that combined the RB and OVRNN models (Fig. 1*D*) and demonstrated that the two alignments can simultaneously occur. Crucially, we found that this occurrence of alignments depends on the excitation/inhibition (E/I) balance of the cortical recurrent RNN. Excessive excitation degrades or even reverses the alignments, resulting in functional impairments. We discuss implications of these results for the causes and symptoms of schizophrenia.

## Materials and Methods

### Outline of the model operation

In OVRNN-RB (Fig. 1*D*), the RNN units’ activities at next time-step were determined by the weighted sum of inputs from the observation units (encoding cue and reward sensations) and the RNN units themselves transformed by a nonlinear input–output function (Eq. 1). The RNN units activate the striatal units through the corticostriatal weights (Eq. 2). The DA units receive inputs from multiple rewards through fixed reward-DA weights and the striatal units through the striatum-DA/BGO (basal ganglia output) weights to calculate/encode multidimensional TD-RPE (Eq. 3). The multi-dim-TD-RPE-encoding DA signals are used in the update of the striatum-DA/BGO weights (Eq. 4) and also sent to the striatal and RNN units via fixed DA-striatum and DA-cortex weights and used in the updates of the corticostriatal weights (Eq. 5) and the weights onto the RNN units (referred to as the RNN weights; Eq. 6), respectively.

The main description of the model architecture, as well as those of the simulations and analyses that we conducted, is placed in the Results, and the figures other than Figures 1 and 13 are also placed in the Results. The following sections of the Materials and Methods describe technical details. Major parameters were set as shown in Table 1.

**Table 1.** Major parameters

Number of the RNN units	40
Number of the striatal units	10 (except for Fig. 7) or 2 (Fig. 7)
Number of the DA units	2 (except for Fig. 4), 3 (Fig. 4*A–D*), or 5 (Fig. 4*E–G*)
Time discount factor *γ*	0.8 (except for Fig. 12*D–F*) or 0.7 (Fig. 12*D–F*)
Learning rate for striatum-DA *α*_SD_	0.03 except for Fig. 11
Learning rate for RNN-striatum *α*_CS_	0.03
Learning rate for RNN weights *α*_RNN_	0.1 (except for after 3,200 trials in Fig. 8*I–L*)

### Online value-RNN with reward bases (OVRNN-RB)

**Figure 2.**
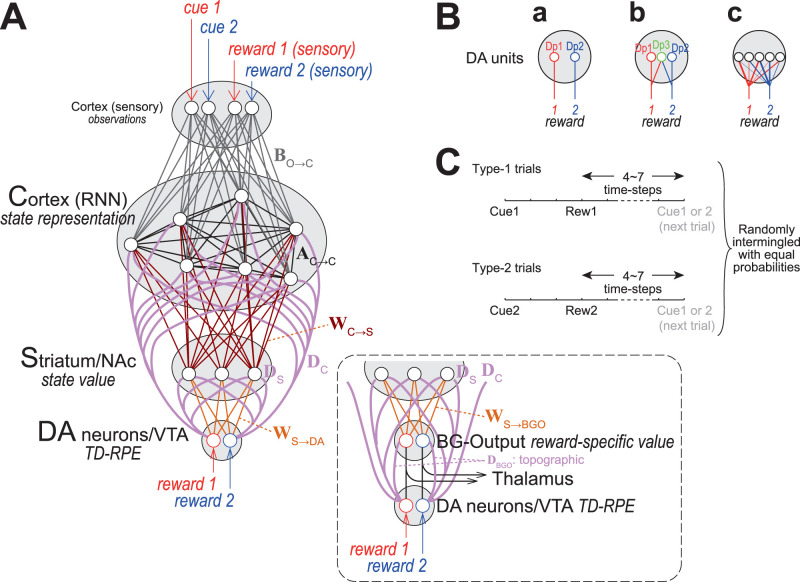
Schematic diagrams of the model and the task. ***A***, Schematic diagram of OVRNN-RB with a simple setting that there are two DA units, each of which receives input exclusively from one of two rewards. Right-bottom inset, A more elaborate diagram of the model, in which the striatal units project to the basal-ganglia output (BGO) units (internal segment of the globus pallidus and substantia nigra pars reticulata), which then project to the thalamus and also the DA units (potentially through axon collaterals; Tepper et al., 1995; Tepper and Lee, 2007). Assuming topographic projections between the DA units and the BGO units (i.e., the *ℓ*-th DA unit projects to the *ℓ*-th BGO unit and vice versa), the same equations mapped onto the original diagram can also be mapped onto this elaborate diagram. ***B***, Different settings about DA units. ***a***, The simple setting as in ***A***. ***b***, An extended setting, where an additional DA unit (Dp3) receives inputs evenly from both rewards. ***c***, The general setting, where multiple DA units receive inputs from the two rewards with fixed random weights. ***C***, Schematic diagram of the simulated behavioral task.

We constructed OVRNN-RB by combining OVRNN, specifically its version with random feedback and biological constraints (defined as “oVRNNrf-bio” in Tsurumi et al., 2025) and RB (Millidge et al., 2024). OVRNN-RB consists of the observation units (modeling sensory cortex), an RNN (modeling prefrontal/association cortex), striatal units, and DA units (Fig. 2*A*). As in OVRNN (Tsurumi et al., 2025), each unit was assumed to represent a population of neurons, and single time-step was assumed to correspond to several hundreds of milliseconds, which are similar to the time scales of certain types of short-term synaptic plasticity (Wang et al., 2006; Mongillo et al., 2008; Morishima et al., 2011) and the behavioral time-scale synaptic plasticity [Bittner et al., 2017; Caya-Bissonnette et al., 2023; see Tsurumi et al. (2025) for detailed discussion for these] and also the time-scale of the temporal-difference(TD)-like computation of striatum-DA circuit (Campbell et al., 2025).

The activities of the observation units at time-step *t*, ***o***(*t*) = (*o_h_*(*t*)) (*h* = 1, …, 4), encoded the presence of a cue or a reward. Specifically, *o*_1_(*t*) or *o*_2_(*t*) became 1 when a cue, Cue 1 or Cue 2, was presented, respectively, while *o*_3_(*t*) or *o*_4_(*t*) became 1 when a reward, Rew 1 or Rew 2, was obtained, respectively, and at other time-steps *o_h_*(*t*) was set to 0.

The activities of the RNN units, ***x***(*t*) = (*x_j_*(*t*)) (*j* = 1, …, 40), were determined depending on the activities of themselves and the observation units at the previous time-step:

x(t+1)=f(Ax(t)+Bo(t)),(1)

where **A** = (*A_ij_*) was the strength of the recurrent connection from *x_j_* to *x_i_* and **B** = (*B_ih_*) was the strength of the feedforward connection from *o_h_* to *x_i_*. *f*(*z*) = 1/(1 + exp(−*z*)) was a sigmoidal function that represented the neuronal input–output relation.

The activity of the striatal units, ***y***(*t*) = (*y_k_*(*t*)) [*k* = 1, 2 (for Fig. 7) or 1,…, 10 (otherwise)], were determined by the following:

$$y(t)=W_{\mathrm{CS}}x(t),\;(2)$$

where **W**_CS_ = (*W*^CS^*_ki_*) was the cortex (RNN)-striatum weight from *x_i_* to *y_k_*.

The activities of the DA units, ***δ***(*t*) = (*δ_ℓ_*(*t*)) [*ℓ* = 1, 2 (except for Fig. 4) or 1, 2, 3 (for Fig. 4*A–D*) or 1,…, 5 (for Fig. 4*E–G*)], were determined by the following:

$$\delta (t)=C_{\mathrm{RD}}r(t)+\gamma W_{\mathrm{SD}}y(t+1)-W_{\mathrm{SD}}y(t),\;(3)$$

where ***r***(*t*) = (*r_m_*(*t*)) (*m* = 1, 2) encoded two rewards: *r*_1_(*t*) or *r*_2_(*t*) became 1 when Rew 1 or Rew 2 was obtained, respectively, and otherwise *r_m_*(*t*) = 0. **C**_RD_ = (*C*^RD^*_ℓm_*) was the fixed reward-DA weight from *r_m_* to *d_ℓ_* and was set to the following:

$$C_{11}^{\mathrm{RD}}=C_{22}^{\mathrm{RD}}=1\;\mathrm{and}\;C_{12}^{\mathrm{RD}}=C_{21}^{\mathrm{RD}}=0\;(\mathrm{except}\;\mathrm{for}\;\mathrm{Fig}.\;4),$$

$$C_{11}^{\mathrm{RD}}=C_{22}^{\mathrm{RD}}=1,C_{12}^{\mathrm{RD}}=C_{21}^{\mathrm{RD}}=0,\mathrm{and}\;C_{31}^{\mathrm{RD}}=C_{32}^{\mathrm{RD}}=0.5\;(\mathrm{for}\;\mathrm{Fig}.\;4A-D),$$

or each *C*^RD^*_ℓm_* was set to a pseudo-uniform random number on [0 1] (for Fig. 4*E–G*). **W**_SD_ = (*W*^SD^*_ℓk_*) was the striatum-DA weight from *y_k_* to *δ_ℓ_*, and *γ* was the time discount factor and was set to 0.8 except for the simulations shown in Figure 12*D–F*, for which *γ* was set to 0.7. As such, ***δ***(*t*) encoded the multidimensional TD-RPE.

### Update rules of OVRNN-RB

The striatum-DA weight **W**_SD_ = (*W*^SD^*_ℓk_*) was initialized to 0 and updated according to the following:

$$W_{\ell k}^{\mathrm{SD}}\leftarrow \max(0,W_{\ell k}^{\mathrm{SD}}+\alpha_{\mathrm{SD}}\delta_{\ell }(t)y_{k}(t)),\;(4)$$

where max(*z*_1_, *z*_2_) returned the larger one of *z*_1_ and *z*_2_ (i.e., *W^SD^_ℓk_* was constrained to be non-negative) and *α*_SD_ was the learning rate and was set to 0.03 except for Figure 11, for which *α*_SD_ was 0.03 until 1,000th trial, linearly decreased from 0.03 to 0 from 1,001th trial to 2,000th trial, kept at 0 from 2,001th to 3,000th trial, and linearly increased from 0 to 0.03 from 3,001th trial to 4,000th trial.

The cortex (RNN)-striatum weight **W**_CS_ = (*W*^CS^*_ki_*) was also initialized to 0 and updated according to the following:

$$W_{ki}^{\mathrm{CS}}\leftarrow \max(0,W_{ki}^{\mathrm{CS}}+\alpha_{\mathrm{CS}}{\left(D_{S}\delta (t)\right)}_{k}x_{i}(t)),\;(5)$$

where the max operation ensured that *W*^CS^*_ki_* was also constrained to be non-negative. **D**_S_ = (*D*^S^*_kℓ_*) was the fixed DA-striatum weight, and (**D**_S_***δ***(*t*))*_k_* indicates the *k*-th element of **D**_S_***δ***(*t*). *α*_CS_ was the learning rate and was set to 0.03.

**Figure 3.**
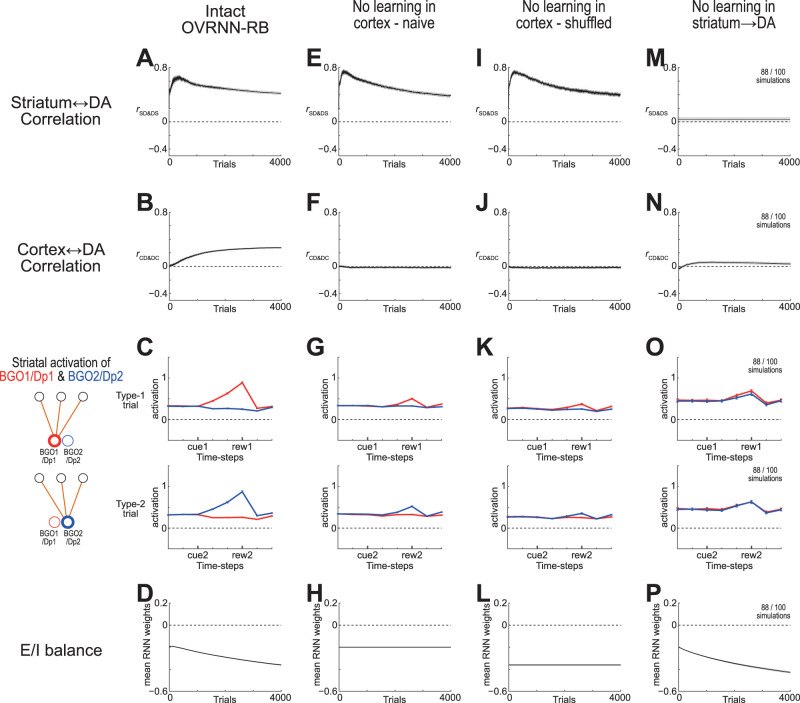
Behavior of OVRNN-RB with two DA units. ***A–D***, Results for the intact OVRNN-RB: ***A***, ***B***, Across-trial evolution of the correlation coefficient between the striatum-DA weights and DA-striatum weights (*r*_SD&DS_; ***A***) or between the cortex (RNN)-striatum-DA connections (product of the cortex-striatum weights and the striatum-DA weights) and the DA-cortex (RNN) weights (*r*_CD&DC_; ***B***); ***C***, Striatal activations of the BGO/DA units (red: BGO1/Dp1, blue: BGO2/Dp2; i.e., product of the striatum-DA weights and the striatal units’ activities) in the last type-1 trial (top) and type-2 trial (bottom) within 4,000 trials [in arbitrary unit (a.u.)]; ***D***, Across-trial evolution of the E/I balance, i.e., the mean of the weights onto the RNN units (in a.u.). For all of ***A–D***, the averages over 100 simulations were shown as thick black lines, and ±SEM were shown as thin gray lines (***A***, ***B***, ***D***) or error bars (***C***; although almost invisible because SEM was small). ***E–H***, Results for a variant model with naive untrained RNN, in which update of the weights onto the RNN units was omitted while all the other settings, including the initializations of the variables, were unchanged. ***I–L***, Results for another variant model with shuffled untrained RNN, in which update of the weights onto the RNN units was omitted and the weights onto the RNN units were initialized to the values that were randomly shuffled from the weights in the leaned OVRNN-RB model at 4,000th trial. ***M–P***, Results for yet another variant model, in which learning of the striatum-DA weights was omitted and those weights were instead fixed to be random values. Twelve out of 100 simulations in which the striatal activation of BGO/DA units became excessively high (>2) were omitted from plotting.

**Figure 4.**
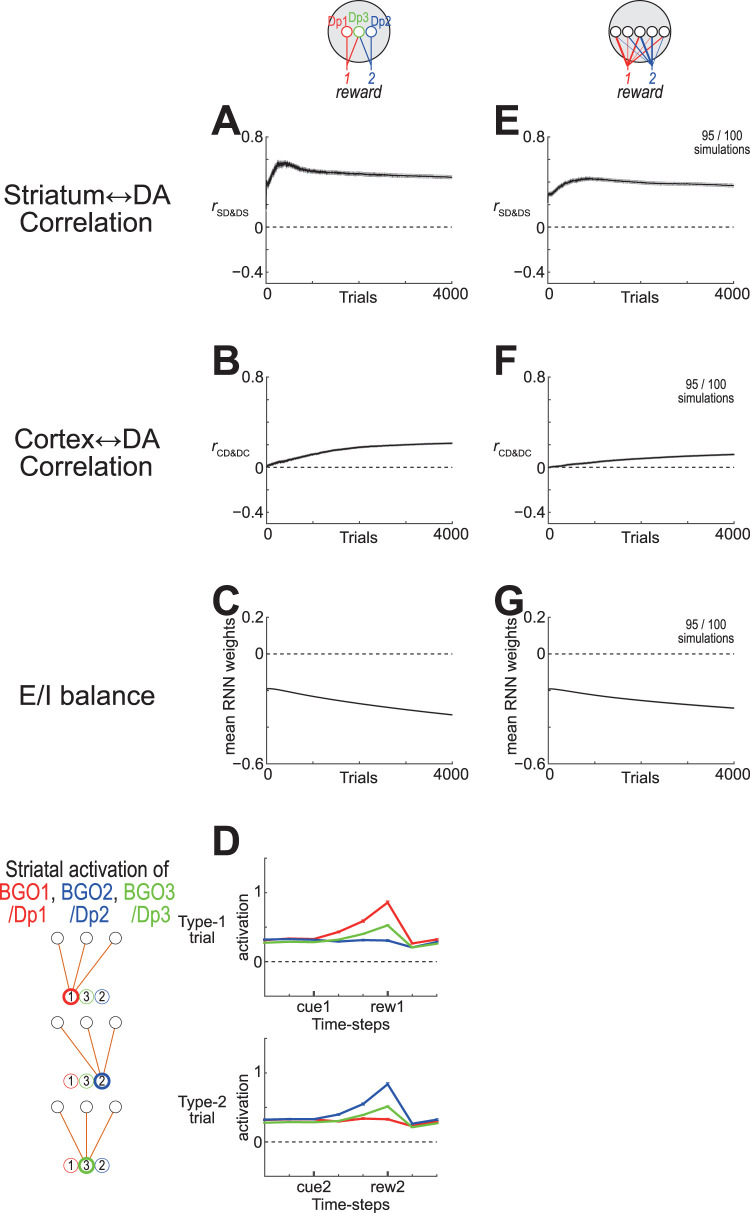
Behavior of OVRNN-RB with more than two DA units. ***A–D***, Case with three DA units: Dp1 and Dp2 receive inputs exclusively from Reward 1 and Reward 2, respectively, and Dp3 receives inputs evenly from both rewards. In ***D***, the green lines indicate the striatal activation of BGO3/Dp3 units. ***E–G***, Case with five DA units, which receive inputs from the two rewards with fixed random weights. The number on the top-right indicates the number of plotted simulations (out of 100 simulations) in which learning failure (weights returning to **0**) did not occur after 100-th trial (same applied to Figs. 5, 12).

**Figure 5.**
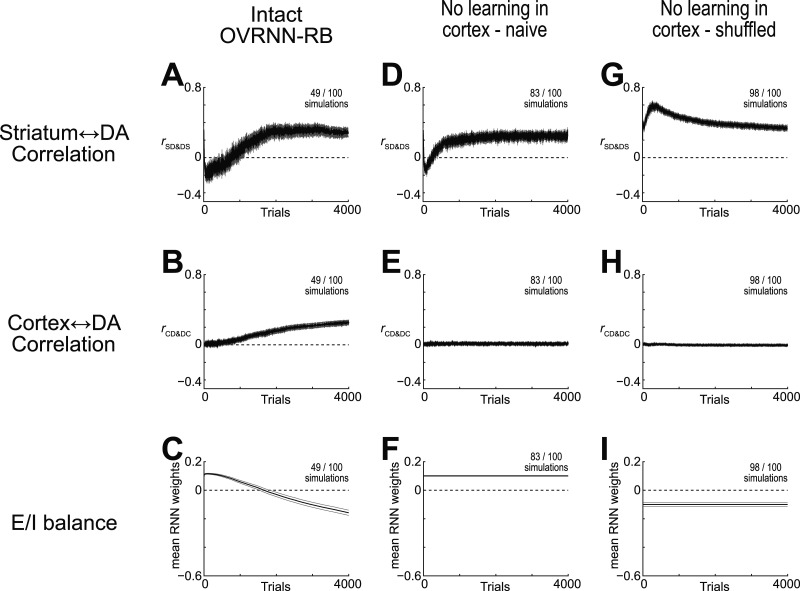
Behavior of OVRNN-RB with two DA units, with the E/I balance was initialized to be excitation-dominant. ***A–F***, Results for the intact OVRNN-RB (***A–C***) or the model with naive untrained RNN (***D–F***) with the mean weight onto the RNN units was initialized to 0.1 (instead of −0.2 as done in Figs. 3, 4). ***G–I***, The model with shuffled untrained RNN, whose weights were shuffled from the leaned intact OVRNN-RB with excitation-dominant initialization (i.e., those shown in ***A–C***) and fixed.

The recurrent and feedforward connection strengths **A** = (*A_ij_*) and **B** = (*B_ih_*) were initialized to pseudo standard normal random numbers plus an offset (initial mean RNN weight), which was set to −0.2 [inhibition-dominant initialization: Figs. 3, 4, 7*B*,*F*,*H*, 8*E–L*, 10*B–D*,*H–J* (left), 11, 13*A*], 0.1 (excitation-dominant initialization: Figs. 5, 7*C*,*G*,*I*, 12), or 0.2 [more excitation-dominant initialization: Figs. 8*A–D*, 10*E–J* (right), 13*B*], and updated at every time-step as follows:

$$(\mathrm{when}\;x_{i}(t)\leq 0.5),$$

$$A_{ij}\leftarrow A_{ij}+\alpha_{\mathrm{RNN}}(D_{C}\delta (t){)}_{i}x_{j}(t-1)x_{i}(t)(1-x_{i}(t)),$$

$$B_{ih}\leftarrow B_{ih}+\alpha_{\mathrm{RNN}}(D_{C}\delta (t){)}_{i}o_{h}(t-1)x_{i}(t)(1-x_{i}(t)),$$

$$(\mathrm{when}\;x_{i}(t)>0.5),$$

$$A_{ij}\leftarrow A_{ij}+0.25\alpha_{\mathrm{RNN}}(D_{C}\delta (t){)}_{i}x_{j}(t-1),$$

$$B_{ih}\leftarrow B_{ih}+0.25\alpha_{\mathrm{RNN}}(D_{C}\delta (t){)}_{i}o_{h}(t-1),\;(6)$$

where **D**_C_ = (*D*^C^*_iℓ_*) was the fixed DA-cortex (RNN) weight and (**D**_C_***δ***(*t*))*_i_* indicates the *i*-th element of **D**_C_***δ***(*t*). *α*_RNN_ was the learning rate and was set to 0.1 except for the time-steps after 3,200 trials in Figure 8*I–L*, for which *α*_RNN_ was set to 0.2 for *i* with the *i*-th element of **D**_C_***δ***(*t*) ≥ 0 and 0.05 for other *i* [i.e., doubled when total DA received by the postsynaptic unit was positive and halved when it was negative (manipulation of learning rate bias)]. The dependence on *x_i_*(*t*) (i.e., postsynaptic activity) was taken from the OVRNN model (Tsurumi et al., 2025), where it was modified from the original non-monotonic dependence [i.e., *x_i_*(*t*)(1 − *x_i_*(*t*)) for arbitrary *x_i_*(*t*)] derived from gradient-descent calculation [more specifically, from the derivative of the input–output function *f*(*z*) = 1 / (1 + exp(−*z*))] so as to be monotonic + saturation that would be more bioplausible. For the time-steps after 3,200 trials in Figure 8*E–H*, 0.0002 was added to each of *A_ij_* and *B_ih_* at every time-step (manipulation of excitation increase). Also, for the time-steps after 2,000 trials in Figure 11, 0.0001 was added to each of *A_ij_* and *B_ih_* at every time-step (manipulation of excitation increase).

Each element of the fixed DA-striatum weight **D**_S_ = (*D*^S^*_kℓ_*) was set to a pseudo-uniform random number on [0 1], except for the cases shown in Figure 7 where *D*^S^_11_ = *D*^S^_22_ = 0.25 and *D*^S^_12_ = *D*^S^_21_ = 0. Each element of the fixed DA-cortex (RNN) weight **D**_C_ = (*D*^C^*_iℓ_*) was set to a pseudo-uniform random number on [0 1].

### Simulation of the behavioral task

We simulated a task of multiple cue–reward associations (Fig. 2*C*), with two cues, Cue1 and Cue2, and two rewards, Rew1 and Rew2. There were two types of trials, which were randomly intermingled with equal probabilities. In type-1 trials, Cue1 was presented and three time-steps later Rew1 was obtained, whereas in type-2 trials, Cue2 was presented and three time-steps later Rew2 was obtained. The cue or reward time-step marked in the figures was defined to be the time-step when the RNN received the cue or reward observation, respectively: if *o_h_*(*t*) = 1 at time *t*, *t* + 1 was defined to be a cue or reward time-step, respectively. Intertrial interval (ITI) was set to 4, 5, 6, or 7 time-steps with equal probabilities. For each model and each condition, we conducted 100 or 1,000 (for Figs. 9*Cg*,*h*, 12) simulations (with different pseudo-random numbers).

### Simulations with fixed state representation and motivational impairments due to anti-alignment

We also examined a model with fixed state representation, FR-RB, to compare with OVRNN-RB in which state representation was adaptively learned. FR-RB had a similar structure to that of OVRNN-RB except that activities of cortical units were determined directly (manually) by the authors according to the following assumptions rather than determined through the RNN dynamics: recurrent connections and connections from observation units were not explicitly modeled in FR-RB and so we used the name of cortical units rather than RNN units for FR-RB. Each time-step of each trial type of the task (including the ITI) was assumed to be represented by specific activation of four cortical units, which were randomly selected out of in total 40 units and fixed for each time-step of each trial type in each single simulation. All the cortical units were assumed to commonly have baseline persistent activity, which was set to *x*_base_ = 0 (for Fig. 9*Aa*,*B–E*), 0.25 (Fig. 9*Ab*), 0.5 (Fig. 9*Ac*), or 0.75 (Fig. 9*Ad*). The randomly selected four units for each time-step of each trial type were assumed to have additional activities, each of which were drawn from the pseudo-uniform distribution over (*x*_base_ 1).

The numbers of striatal and DA units were set to 10 and 2, respectively. The two DA units, Dp1 and Dp2, were assumed to receive reward input exclusively from reward 1 and reward 2, respectively, as in the simplest setting of OVRNN-RB (Fig. 2*Ba*). The cortex-striatum weights were assumed to be learned in the same way as in OVRNN-RB. The striatum-DA weights **W**_SD_ = (*W*^SD^*_ℓk_*) were also learnable in simulations for Figure 9*A*, while they were predetermined (and fixed) in simulations for Figure 9*B–E* in either of two different ways: in the striatum ↔ DA alignment condition, **W**_SD_ = (*W*^SD^*_ℓk_*) was assumed to be a transpose of the fixed random DA → striatum weight matrix **D**_S_ = (*D*^S^*_kℓ_*), whereas in the striatum ↔ DA anti-alignment condition, **W**_SD_ = (*W*^SD^*_ℓk_*) was assumed to be a transpose of *D*^S^*_k_*_(3 − _*_ℓ_*_)_, which was **D**_S_ with two columns swapped.

We further examined the effects of reward identity-specific motivational saliency. We assumed that when the motivational salience index of reward *i* (*i* = 1, 2) was *μ_i_*, the activities of the striatal units ***y***(*t*) = (*y_k_*(*t*)) were multiplied by the following:

$$1+D_{S}(\mu_{1}\mu_{2}{)}^{T}.$$

This formula means that when *μ_i_* is positive[/negative; i.e., reward *i* has a high(/low) motivational salience], the activities of striatal units that are projected by Dp*_i_* unit, which receives reward input exclusively from reward *i*, are enhanced(/suppressed) by the degrees proportional to (*μ_i_* times the weight from Dp*_i_*), presumably through the effects of tonic DA as considered in the RB model and its preceding models [Collins and Frank, 2014; Möller and Bogacz, 2019; Millidge et al., 2024; notably, we incorporated the effect of tonic DA on motivational modulation only through *μ_i_* here and in the schematic illustration described below, and ***δ***(*t*) always encoded multidimensional TD-RPE (but not motivational modulation) based on the suggestions that diverse patterns of DA encode TD-RPE (Gershman et al., 2024; Kato and Morita, 2025)]. We examined two conditions with (*μ*_1_, *μ*_2_) = (2, −1) (reward 1 had a high salience and reward 2 had a low salience) and (−1, 2) (reward 1 had a low salience and reward 2 had a high salience) and calculated the results for 100 simulations for each condition for each of the FR-RB models with striatum ↔ DA alignment and striatum ↔ DA anti-alignment.

### Simulated inputs to the striatum in a probabilistic version of the task

We examined the behavior of OVRNN-RB (40 RNN units; 10 striatal units; 2 DA units; *C*^RD^_11_ = *C*^RD^_22_ = 1 and *C*^RD^_12_ = *C*^RD^_21_ = 0) with the RNN weights initialized to either inhibition(I)-dominant [−0.2, modeling healthy-control participants (HC)] or excitation(E)-dominant [0.2, modeling schizophrenia patients (SZ); 100 simulations for each] in a probabilistic version of the task, in which reward was probabilistically obtained in two-thirds (66.7%) of trials. We calculated the cortical and DA inputs to the striatal units, ***y***(*t*) = **W**_CS_***x***(*t*) and **D**_S_***δ***(*t *− 1), respectively, and the sums of their elements:

$$\mathrm{In}p_{\mathrm{CS}}(t)=(11\ldots 1)W_{\mathrm{CS}}x(t),$$

$$\mathrm{In}p_{\mathrm{DS}}(t)=(11\ldots 1)D_{S}\delta (t-1).$$

We then subtracted the average across the last 100 trials within 4,000 trials from each of them to obtain the normalized input:

normInpCS(t)=InpCS(t)−meanofInpCS(t)acrossthelast100trials,

normInpDS(t)=InpDS(t)−meanofInpDS(t)acrossthelast100trials.

We calculated their sum: _norm_Inp_CS_(*t*) + _norm_Inp_DS_(*t*), which could be considered as a proxy of striatal BOLD signal. We obtained _norm_Inp_CS_(*t*) and _norm_Inp_DS_(*t*) at the second last trial (used for Fig. 10*H*), second last rewarded trial (for Fig. 10*D*, left; 10*G*, left; and 10*I*,*J*), and second last not-rewarded trial (for Fig. 10*D*, right; 10*G*, right; and 10*I*,*J*) within 4,000 trials for each of the two trial (cue/reward) types and took averages across the trial types. Then, across-simulation mean and SEM of their averaged values for the post-cue to reward time-steps (“reward anticipation” phase), values at the time-step just after reward (“outcome-early” phase), and values at two time-steps from reward (“outcome-late” phase) were plotted and analyzed.

### Schematic illustration of the reward-specific modulation of value/motivation through alignment

**Figure 6.**
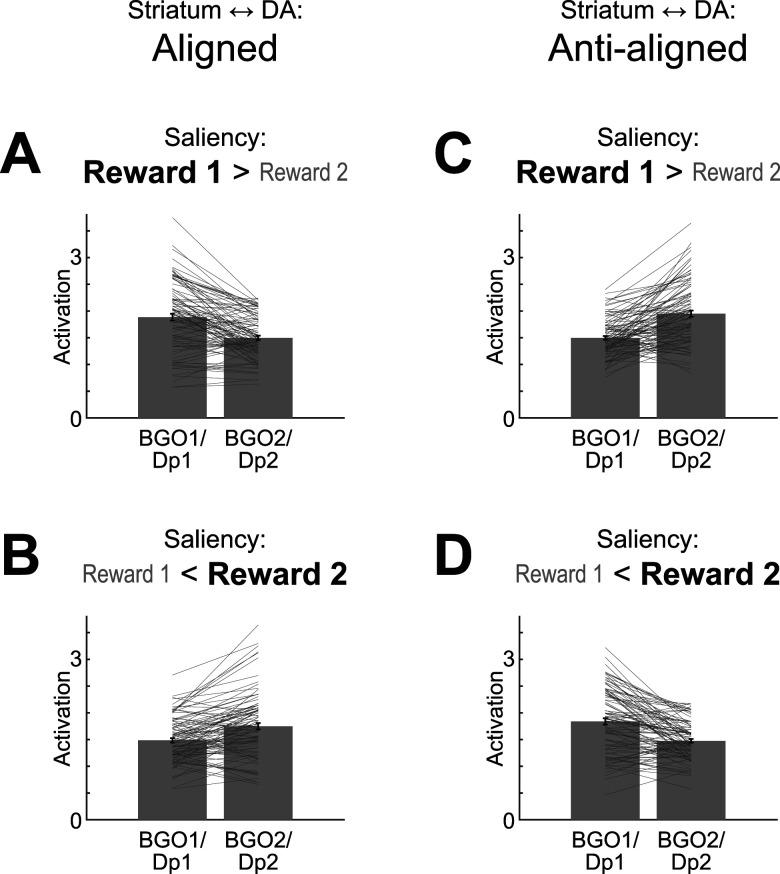
Schematic illustration of the reward-specific modulation of value/motivation achieved by striatum ↔ DA alignment and its impairment (reversal) by striatum ↔ DA anti-alignment. ***A***, ***B***, In the case with striatum ↔ DA alignment, when reward 1 has high motivational salience and reward 2 has low salience (***A***), the activation of BGO1/Dp1 (reflecting reward 1-specific value) becomes higher than the activation of BGO2/Dp2 (reflecting reward 2-specific value), whereas when reward 2 has higher salience (***B***), the activation of BGO2/Dp2 becomes higher. ***C***, ***D***, In the case with striatum ↔ DA anti-alignment, when reward 1 has higher salience (***C***), the activation of BGO2/Dp2 becomes higher, whereas when reward 2 has higher salience (***D***), the activation of BGO1/Dp1 becomes higher.

For the schematics in Figure 6, we assumed that striatal activities ***y*** = (*y_k_*) were multiplied by 1 + **D**_S_(*μ*_1_ *μ*_2_)^T^, where **D**_S_ was the random DA → striatum weights and *μ_i_* was 2 or −1 when reward *i* had high or low motivational salience, respectively, through the effects of tonic DA from Dp*_i_* unit. We assumed that each *y_k_* was a uniform pseudo-random number on [0 1] and calculated activations of BGO1/Dp1 and BGO2/Dp2 units (**W**_SD_***v***) in the cases with striatum ↔ DA alignment, where **W**_SD_ = (*W*^SD^*_ℓk_*) was a transpose of **D**_S_ = (*D*^S^*_kℓ_*) and striatum ↔ DA anti-alignment, where **W**_SD_ was a transpose of *D*^S^*_k_*_(3 − _*_ℓ_*_)_, which was **D**_S_ with two columns swapped, 100 times each with different pseudo-random numbers.

### Analyses

Alignments of the forward connections to the feedback connections were evaluated by their correlations. Specifically, alignment of the striatum-DA weights to the DA-striatum weights was quantified, at every trial, by the correlation coefficient between the elements of **W**_SD_ and the elements of **D**_S_. Alignment of the cortex (RNN)-striatum-DA connections to the DA-cortex (RNN) weights was quantified, at every trial, by the correlation coefficient between the elements of **W**_SD_**W**_CS_ and the elements of **D**_C_. Correlation coefficient was calculated by corrcoef function of MATLAB.

Standard error of the mean (SEM) shown in the figures was approximated by SD (standard deviation)/√N (number of samples). Simulations were conducted by using MATLAB, and pseudo-random numbers were implemented by using rand, randn, and randperm functions. For statistical comparisons, Wilcoxon rank sum test was conducted by using wilcox.exact in the package of exactRankTests in R.

### Code accessibility

The codes for simulations and analyses are available at GitHub: https://github.com/kenjimoritagithub/OVRNN-RB.

## Results

### Online value-RNN-reward bases (OVRNN-RB) model

We constructed a model of the mesocorticostriatal system, by integrating two recent models: the online value-RNN (OVRNN; Tsurumi et al., 2025) and the reward bases (RB; Millidge et al., 2024). OVRNN, a biologically plausible version of the original value-RNN (Hennig et al., 2023), consists of an RNN (corresponding to cortex), a readout unit (striatum), and an error unit (DA neurons). The error unit calculates a scalar (i.e., one-dimensional) temporal-difference reward-prediction-error (TD-RPE), which is sent to the striatal readout and also to the cortical RNN via fixed random weights. In the RNN-readout (corticostriatal) weights, state value is learned, as in the standard reinforcement learning (RL) model of basal ganglia (Doya, 2000). In the meantime, in the cortical RNN, state representation appropriate for the current task is learned, by virtue of an “alignment” (cf. Lillicrap et al., 2016; Murray, 2019; Bellec et al., 2020) of the RNN-readout (corticostriatal) weights to the fixed random error-feedback (mesocortical) weights.

On the other hand, RB (Millidge et al., 2024), developed in reference to previous studies (Collins and Frank, 2014; Keramati and Gutkin, 2014; Möller and Bogacz, 2019; Wärnberg and Kumar, 2023), consists of cortical inputs, striatal value units, and DA error units. The DA units are heterogeneous as they receive differential inputs from multiple different rewards, encoding multidimensional TD-RPEs as a whole. The TD-RPEs are sent to the striatal units via fixed random weights and used for the training of corticostriatal weights, as well as the training of striatum-DA weights. Through learning, an “alignment” of the striatum-DA weights to the fixed random DA-striatum weights occurs: for instance, if a striatal unit receives a strong feedback from a DA unit that is strongly activated by a particular reward, the forward connection from this striatal unit to this DA unit becomes also strong. This alignment enables reward-specific value encoding and motivational control. Specifically, when that particular reward is highly desired (e.g., food when hungry), raised tonic DA (cf. Niv et al., 2007; Collins and Frank, 2014; Möller and Bogacz, 2019) from that DA unit can amplify, via physiological modulation, the input from the “corresponding” striatal unit, resulting in a specific amplification of the value of that reward (Millidge et al., 2024).

As such, OVRNN deals with cortical RNN dynamics and its training by TD-RPE but not heterogeneity of rewards and TD-RPE signals, whereas RB deals with the latter but not the former. We constructed a model, OVRNN-RB (Fig. 2*A*,*B*), which combined OVRNN and RB, incorporating both heterogeneous TD-RPEs and training of cortical RNN by them. As mentioned above, learning of OVRNN is ensured by the alignment of the RNN-downstream weights to the DA-RNN (mesocortical) weights, while learning of RB is achieved through the alignment of the striatum-DA weights to the DA-striatum (mesostriatal) weights. When OVRNN and RB are combined, whether these two alignments can both occur is nontrivial, especially given that the RNN-downstream weights become more complex than those in OVRNN because there are now multiple striatal and DA units. As in the biologically most plausible version of OVRNN (“oVRNNrf-bio” in Tsurumi et al., 2025), we imposed biological constraints that the activity of RNN units and the weights of the RNN-striatum, striatum-DA, DA-RNN, and DA-striatum connections were non-negative and also the dependence of the update (plasticity) of the RNN weights on the postsynaptic activity was monotonic(+saturation). In addition, because it was shown that in OVRNN the mean of the weights onto the RNN units (hereafter referred to as the RNN weights) became negative (i.e., inhibition-dominance in the E/I balance) through learning (Tsurumi et al., 2025), here we initialized the RNN weights to be negative on average. We calculated the correlation coefficient *r* between the forward and feedback connections as an index of alignment (similarly to the previous study proposing the RB model; Millidge et al., 2024), referring to *r* > 0 as aligned and *r* < 0 as anti-aligned (comparison of *r* and cosine similarity, which is another popular index of alignment, is presented in the Discussion).

### Learning of reward-specific representation and value through double alignments

We simulated a task with multiple cue–reward associations (Fig. 2*C*). There were two cues, Cue1 and Cue2, and two rewards, Rew1 and Rew2. There were two types of trials. In type-1 trials, Cue1 was presented, and three time-steps later, Rew1 was obtained, whereas in type-2 trials, Cue2 was presented, and three time-steps later, Rew2 was obtained (single time-step was assumed to correspond to several hundreds of milliseconds: see the Materials and Methods for details). Type-1 trials and type-2 trials were randomly intermingled with equal probabilities, and ITIs were randomly set to 4–7 time-steps. We examined how the cortex (RNN)-striatum-DA connections and striatum-DA weights developed and the system behaved in the two types of trials.

We started with a simple configuration of the OVRNN-RB model where there were two DA units, Dp1 and Dp2, each of which received input exclusively from one of the two rewards, Rew1 and Rew2, respectively (Fig. 2*Ba*). As mentioned above, as a measure of alignment, we calculated the correlation coefficient between the striatum-DA weights and the fixed random DA-striatum weights (denoted as *r*_SD&DS_; Fig. 3*A*) and the correlation coefficient between the cortex (RNN)-striatum-DA connections (i.e., product of the RNN-striatum weights and the striatum-DA weights) and the fixed random DA-RNN weights (denoted as *r*_CD&DC_; Fig. 3*B*). Across-simulation averages of *r*_SD&DS_ rapidly increased and then modestly decreased but remained positive, while *r*_CD&DC_ increased more gradually. These positive correlations indicate that alignments of the cortex-striatal-DA connections and the striatum-DA weights to the mesocortical and mesostriatal weights, respectively, both occurred.

Figure 3*C* shows across-simulation averages of the striatal activations of the Basal-Ganglia-Output (BGO1/BGO2) and DA (Dp1/Dp2) units (i.e., product of the striatum-DA weights and the striatal units’ activities) in the last type-1 trial (Fig. 3*C*, top) and type-2 trial (Fig. 3*C*, bottom) within 4,000 trials, respectively. In type-1 trial, activation of BGO1/Dp1 units encoded the (temporally discounted) state values starting from Cue1 and ending upon Rew1 while activation of BGO2/Dp2 units was largely flat, whereas in type-2 trial, activation of BGO2/Dp2 units encoded the state values from Cue2 to Rew2 while activation of BGO1/Dp1 units was largely flat. These results indicate that reward-specific state representation and value were successfully learned in the model. Crucially, this enables reward-specific motivational control, as in the ancestor RB model (Millidge et al., 2024). Specifically, under a situation where Rew1 (e.g., food) entails a high motivational desirability (i.e., hunger), the enhanced motivation for Rew1 can be encoded as a raised tonic DA from Dp1 unit, which amplifies responses of striatal units that receive strong inputs from Dp1 [not incorporated in the simulations shown in Fig. 3; we will deal with this later (compare Figs. 6, 9*D*,*E*)]. Then, if these striatal units in turns strongly project to Dp1, i.e., if the striatum-DA weights are aligned to the DA-striatum weights, the increase of their responses means a specific amplification of the values from Cue1 to Rew1 (i.e., values preceding food), without amplification of the values from Cue2 to Rew2.

To understand how the learning of the RNN weights contributed to these results, we examined two variant models: naive untrained RNN and shuffled untrained RNN. In both models, update of the RNN weights was omitted while update of the RNN-striatum and striatum-DA weights was kept intact. In the naive untrained RNN variant, RNN weights were initialized in the same manner as in the OVRNN-RB. In the shuffled untrained RNN variant, to initialize the RNN weights, we took the weights of a learned OVRNN-RB model (at 4,000th trial) and randomly shuffled those. Figure 3, *E–G* and *I–K*, shows the results for the two variants. In both, *r*_SD&DS_ rapidly increased, indicating that alignment at the striatum-DA part still occurred, whereas *r*_CD&DC_ remained around 0, indicating no alignment (as expected). Trial-type-specific development of the activation of BGO1/Dp1 or BGO2/Dp2 unit was much poorer than the case of the intact OVRNN-RB model. These results indicate that alignment at the cortex (RNN)-striatum part requires learning of the RNN weights, and it is pivotal for development of reward-specific values when appropriate state representation is not given but needs to be learned in the RNN.

We also examined a third variant, in which update of the striatum-DA weights was omitted while update of the RNN and RNN-striatum weights was kept intact. In this case, in some simulations (12 out of 100), the striatal activation of BGO/DA units became excessively high (>2), indicating a learning failure. Analyzing the remaining simulations, *r*_SD&DS_ remained to be around 0 (Fig. 3*M*) as expected while *r*_CD&DC_ increased but only slightly (Fig. 3*N*), and state values were developed to a certain extent but there was no trial-type/unit-selectivity, also as expected (Fig. 3*O*).

Next, we examined an extended configuration of the OVRNN-RB model where there were three DA units (Fig. 2*Bb*): Dp1 and Dp2 receive exclusive inputs from reward Rew1 and Rew2, respectively, while Dp3 receives inputs evenly from both rewards. As shown in Figure 4*A*,*B*, *r*_SD&DS_ rapidly increased and remained to be positive, and *r*_CD&DC_ gradually increased, similarly to the simpler case without Dp3 unit. As for the striatal activations of BGO/DA units, BGO1/Dp1 and BGO2/Dp2 units were activated in type-1 and type-2 trials, respectively, while BGO3/Dp3 units were activated to an intermediate level in both trial types (Fig. 4*D*). This indicates that reward-specific values were developed also in this model with three DA units. Finally, we examined a more general configuration where there were five DA units, which received fixed random (varied across simulations) inputs from the two reward types (Fig. 2*Bc*). In this model too, both *r*_SD&DS_ and *r*_CD&DC_ became positive (Fig. 4*E*,*F*), indicating the occurrence of double alignments, although in 5 out of 100 simulations, the striatum-DA weights returned to **0** after 100 trials (i.e., learning failed and restarted), and they were omitted from the figures.

### Excessive excitation causes striatum-DA anti-alignment

In OVRNN-RB, the RNN weights were allowed to take both positive and negative values for simplicity. Based on our previous work on OVRNN (Tsurumi et al., 2025), we can expect that the model could be elaborated to a more biologically plausible setting where output weights of each unit can only be either excitatory and inhibitory. In all the simulations/models so far shown, the mean RNN weight started from a negative value because of the aforementioned inhibition-dominant initialization (mean weight: −0.2), and training made the RNN weights further negative (Figs. 3*D*,*P*, 4*C*,*G*; although initially a slight positive shift appeared), unless the update of the RNN weights was omitted (Fig. 3*H*,*L*).

To test how important it was to initialize the RNN with negative average weights, we initialized the RNN weights to be excitation-dominated (i.e., the initial mean RNN weight to be 0.1 instead of −0.2 that was so far assumed). With such excitation-dominated RNNs, there were many instances (51 out of 100 simulations) when learning failed (i.e., the striatum-DA weights returned to **0**) after 100 trials. In the remaining cases where the model successfully learned, *r*_SD&DS_ rapidly decreased to become negative, and then increased to eventually become positive (Fig. 5*A*), while *r*_CD&DC_ slowly increased (Fig. 5*B*). The initial decrease and negative value of *r*_SD&DS_ were also observed in the model with naive untrained RNN with excitation-dominant initialization (Fig. 5*D*). In contrast, such a pattern did not appear (Fig. 5*G*) in the model with untrained RNN, whose weights were shuffled from learned OVRNN-RB, which was initialized to be excitation-dominant but eventually became inhibition-dominant (as shown in Fig. 5*C*).

These results suggest that negative *r*_SD&DS_, i.e., anti-alignment of the striatum-DA weights to the DA-striatum weights (while these weights remained non-negative because we had constrained them to be so) may be caused by E/I imbalance in the cortical RNN, more specifically, excessive excitation (or insufficient inhibition). Such an anti-alignment should impair reward-specific motivational control, because it would mean a decrease in the value of states associated with highly desired rewards while state values leading to other rewards are amplified (e.g., when hungry, states leading to food are lower valued whereas values leading to drink are higher valued, and opposite occurs when thirsty), as illustrated in Figure 6.

### Role of persistent activity

**Figure 7.**
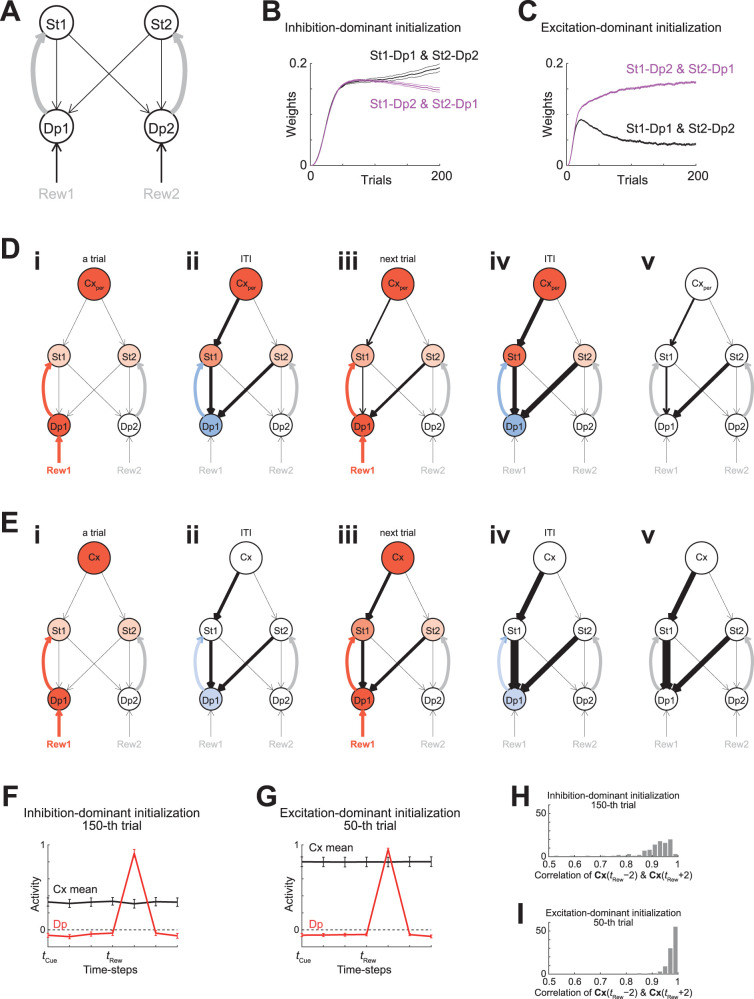
Excessive excitation-induced aberrant persistent activity in the cortex (RNN) causes anti-alignment of the striatum-DA weights to the DA-striatum weights. ***A***, OVRNN-RB with a simple setting, where two DA units (Dp1 and Dp2), activated exclusively by Rew1 and Rew2, project exclusively to two striatal units (St1 and St2), respectively. ***B***, ***C***, Across-trial evolution of the mean strength of St1-Dp1 and St2-Dp2 (black) and St1-Dp2 and St2-Dp1 (magenta) weights in the case of inhibition-dominant (−0.2; ***B***) or excitation-dominant (0.1; ***C***) initialization of the RNN weights. ***D***, Schematic explanation of how cortical aberrant persistent activity (Cx_per_) causes anti-alignment (i.e., St1-Dp1 < St1-Dp2). ***E***, Schematic explanation of how feedback alignment (i.e., St1-Dp1 > St1-Dp2) is formed if the cortical population (Cx) does not have aberrant persistent activity. ***F***, ***G***, The mean activity of the RNN units (black lines) and the activity of the DA unit (corresponding to the reward type of the trial, which varied in individual simulations; red lines) at the 150th trial of the inhibition-dominant case (***F***), where positive feedback alignment was being formed (compare panel ***B***) or at the 50th trial of the excitation-dominant case (***G***), where anti-alignment was being formed (compare panel ***C***). ***H***, ***I***, Across-simulation distributions of the correlation coefficient of the RNN activity pattern at two time-steps before reward (*t*_Rew_ − 2, during task trial) and the pattern at two time-steps after reward (*t*_Rew_ + 2, during ITI) at the 150th trial of the inhibition-dominant case (***H***) or at the 50th trial of the excitation-dominant case (***I***).

To better understand the possible anti-alignment in excitation-dominated network, we conducted simulations using OVRNN-RB with a simplified setting, where two DA units (Dp1 and Dp2), activated exclusively by Rew1 and Rew2, project exclusively to two striatal units (St1 and St2), respectively (Fig. 7*A*), with the RNN initialized to either inhibition-dominant or excitation-dominant. In the case of inhibition-dominant initialization, St1-Dp1 and St2-Dp2 weights became stronger than St1-Dp2 and St2-Dp1 weights (i.e., the striatum-DA weights were aligned to the fixed DA-striatum weights) within 200 trials (Fig. 7*B*; example in Movie 1). In contrast, in the case of excitation-dominant initialization, St1-Dp2 and St2-Dp1 weights became stronger than St1-Dp1 and St2-Dp2 weights, i.e., the striatum-DA weights were anti-aligned to the fixed DA-striatum weights (Fig. 7*C*; example in Movie 2). Generally, positive mean value of weights can affect the activity of RNNs in multiple ways, e.g., the dynamics can become unstable or network reaches a trivial fixed point with persistent activity (Rajan and Abbott, 2006). We hypothesized that emergence of task-irrelevant aberrant persistent activity in excitation-dominated network may underlie the anti-alignment.

**Movie 1.** Example run of OVRNN-RB with a simple setting (two striatal units) in the case of inhibition-dominant (−0.2) initialization of the RNN weights examined in Figure 7*B*. [View online]

**Movie 2.** Example run of OVRNN-RB with a simple setting (two striatal units) in the case of excitation-dominant (0.1) initialization of the RNN weights examined in Figure 7*C*. [View online]

To illustrate this hypothesis, we considered a simplified schematic with a cortical population that is persistently active during task trials and ITIs (Cx_per_), two DA units (Dp1 and Dp2), and two striatal units (St1 and St2; Fig. 7*Di*). This schematic gives an intuition about how learning would shape the striatum-DA connections (i.e., St1 → Dp1, St1 → Dp2, St2 → Dp1, and St2 → Dp2) if they all had the same initial weights.

When reward Rew1 is obtained in a trial, Dp1 encodes positive TD-RPE (Fig. 7*Di*). This causes, in the subsequent ITI (Fig. 7*Dii*), a potentiation of St1 → Dp1 and St2 → Dp1 weights and also potentiates Cx_per_ → St1 but not Cx_per_ → St2 weight via the assumed exclusive Dp1 → St1 connection so that St1 becomes more activated by Cx_per_ than St2. In the ITI, Dp1 tends to encode negative TD-RPE, because TD-RPE = 0 + *γ*·value(*t*) − value(*t *− 1) tends to be negative given that the time discount factor *γ* is smaller than 1 and the value function has not been well learned so that value(*t*) and value(*t* − 1) take similar near-random values. This negative TD-RPE causes, subsequently (Fig. 7*Diii*), a depression (LTD, or depotentiation) of St1 → Dp1 and St2 → Dp1 weights, but crucially, more prominently for St1 → Dp1 than St2 → Dp1 weight because St1 was more activated by Cx_per_ than St2 (the degree of plasticity induction was presumed to depend on the pre-synaptic activity), resulting in weaker St1 → Dp1 weight than St2 → Dp1 weight. In this way, given that the feedback weights have Dp1 → St1 > Dp1 → St2 (=0), the forward weights become St1 → Dp1 < St2 → Dp1, i.e., anti-aligned to the feedback weights.

In the next trial and ITI (Fig. 7*Diii–v*), largely similar things occur. A difference is that Dp1's positive TD-RPE potentiates St1 → Dp1 more prominently than St2 → Dp1 weight because St1 is more active than St2, mildening the previously formed St1 → Dp1 < St2 → Dp1 weight difference (Fig. 7*Div*). However, this positive TD-RPE also further potentiates Cx_per_ → St1 but not Cx_per_ → St2 weight, and St1 becomes even more activated by Cx_per_ than St2 (Fig. 7*Div*) so that negative TD-RPE depresses (or depotentiates) St1 → Dp1 even more prominently than St2 → Dp1 weight (Fig. 7*Dv*). Therefore, in total, St1 → Dp1 weight becomes even weaker than St2 → Dp1 weight; i.e., anti-alignment is enhanced.

Learning in the inhibition-dominated regime, without persistent cortical activity during ITIs, can be similarly understood using a schematic (Fig. 7*E*, Cx indicating cortical population). A crucial difference from the excitation-dominated regime is that, even though negative TD-RPE is generated during ITIs, it does not cause depression (LTD, or depotentiation) of St → Dp or Cx → St weights because the degree of plasticity induction was presumed to depend on the pre-synaptic activity, which is 0 during ITIs. Consequently, positive TD-RPE upon receival of reward Rew 1 potentiates St1 → Dp1, St2 → Dp1, and Cx → St1 (but not Cx → St2) weights (Fig. 7*Ei*,*ii*), and upon receival of Rew1 in the next trial, positive TD-RPE potentiates St1 → Dp1 more than St2 → Dp1 weight because St1 is now more activated than St2 by Cx through the potentiated Cx → St1 weight. In this way, given that the feedback weights have Dp1 → St1 > Dp1 → St2 (=0), the forward weights become St1 → Dp1 > St2 → Dp1, i.e., positively aligned to the feedback weights, achieving the feedback alignment.

To confirm the validity of these schematic descriptions, we returned to the simulations of the simplified setting. We analyzed the activities of the cortical RNN units and the DA units, comparing the 150th trial of the case of inhibition-dominant initialization, where positive feedback alignment was being formed (compare Fig. 7*B*), and the 50th trial of the case of excitation-dominant initialization, where anti-alignment was being formed (compare Fig. 7*C*). The activity of the DA unit (corresponding to the reward type of the trial, which varied in individual simulations) became positive at the post-reward time-step and negative at the other time-steps (including those in the ITI) in both inhibition-dominant and excitation-dominant initialization cases (Fig. 7*F*,*G*, red lines), as conjectured in the schematics.

The mean cortical RNN activity was ∼0.32 in the inhibition-dominant case (Fig. 7*F*, black line) and 0.8 in the excitation-dominant case (Fig. 7*G*). Moreover, the correlation of the RNN activity pattern at two time-steps before reward (during task trial) and the pattern at two time-steps after reward (during ITI) was higher (i.e., closer to 1) in the excitation-dominant case (Fig. 7*I*) than in the inhibition-dominant case (Fig. 7*H*). This suggests that RNN units were more likely to keep similar patterns and levels of persistent activities throughout the task trial and ITI in the excitation-dominant regime than in the inhibition-dominant regime, as we conjectured in the schematics (although persistent activity during ITI did exist even in the inhibition-dominant case, deviating from the schematics). Thus, we consider that the abovementioned schematic explanations are valid at least to a certain extent. Nonetheless, the high mean RNN activity in the excitation-dominant case and the rather high correlation of during-trial and during-ITI activity patterns already in the inhibition-dominant case would be better to be amended in future studies to pursuit further biological plausibility, in particular, sparseness and irregularity of cortical neural activities.

### Excitation/inhibition imbalance induced by different processes causes similar learning impairment

**Figure 8.**
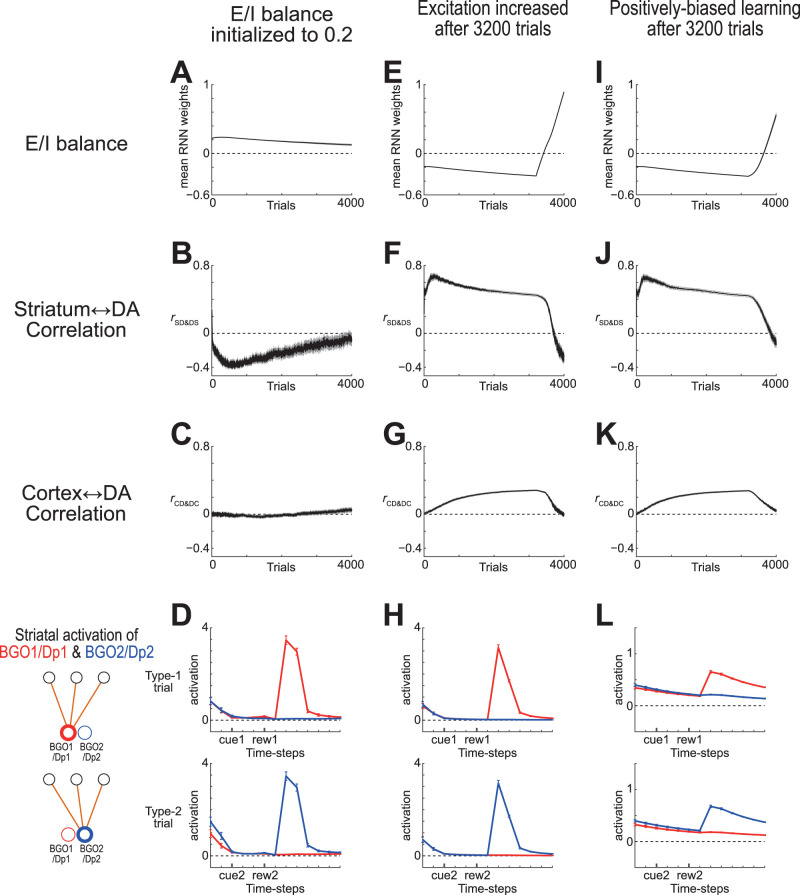
Effects of manipulations that affect the E/I balance. ***A–D***, The E/I balance was initialized to be more excitation-dominant (mean RNN weight = 0.2). ***E–H***, After 3,200 trials, a small positive value (0.0002) was added to each RNN weight at each time-step. ***I–L***, After 3,200 trials, the learning rate for the RNN weights was doubled when total DA received at postsynaptic RNN unit [i.e., product of RPE and the DA-cortex (RNN) connections] was positive and halved when it was negative. In this figure, at each trial, simulations where correlation coefficient could not be calculated (because all the elements of either weight vector had a same value such as 0) were omitted from plotting, while simulations with learning failure after 100 trials were included (different from Figs. 4, 5, 12).

In the simulations shown in Figure 5*A–C* where the RNN weights were initialized to be excitation-dominant, more specifically, 0.1 on average, even though anti-alignment of the striatum-DA and DA-striatum weights (i.e., negative *r*_SD&DS_) occurred in the early phase, *r*_SD&DS_, as well as *r*_CD&DC_, eventually became positive, indicating successful learning, and the mean RNN weights eventually became negative (in the simulations shown in the figure without learning failure after 100 trials). We examined learning in the case where the RNN weights were initialized to be more excitation-dominant, in particular, 0.2 on average. As shown in Figure 8*A–C*, in this case, *r*_SD&DS_ remained to be negative and *r*_CD&DC_ remained to be around 0 for a long duration, indicating the persistence of learning impairment, although they still continued to increase slowly while the mean RNN weights continued to decrease.

Next, in order to simulate situations where the E/I balance changes upon time, we examined the cases where the RNN weights were initialized to be inhibition-dominant and learning proceeded properly but later on manipulation was added so that the RNN weights were shifted to the positive direction. We examined two sorts of manipulations. The first one was a direct positive shift of each RNN weight, which could abstractly model glutamate or GABA-related factors. Specifically, a small positive value (0.0002) was added to each RNN weight at each time-step. The second manipulation was a bias in the learning rate for the update of the RNN weights, which was based on the suggestions that changes in the DA level could cause learning rate biases, specifically, larger learning rate for positive than negative, or negative than positive, RPE (Frank et al., 2004; Collins and Frank, 2014; Pinto and Uchida, 2023), although such suggestions were made about corticostriatal weights rather than about intracortical weights. Specifically, we considered a manipulation where the learning rate was doubled when total DA received at postsynaptic RNN unit (i.e., product of RPE and the DA-cortex (RNN) connections) was positive and halved when it was negative.

Figure 8, *E–G* and *I–K*, shows the results of simulations, in which either of the two manipulations was applied after 3,200 trials. Both manipulations shifted the mean RNN weights to the positive direction, as expected (Fig. 8*E*,*I*). The correlation between the striatum-DA and DA-striatum weights (*r*_SD&DS_) decreased and eventually became negative (Fig. 8*F*,*J*), indicating that their alignment, necessary for reward-specific motivational control, was degraded and eventually reversed. The correlation between the cortex (RNN)-striatum-DA connections and DA-cortex (RNN) weights (*r*_CD&DC_) also decreased and approached around 0 (Fig. 8*G*,*K*), indicating that their alignment was also degraded. These results demonstrated that even after learning of OVRNN-RB properly proceeded and both alignments were formed, the alignments were degraded by either a direct positive shift of the RNN weights or a positive bias in the learning rate for their update.

Figure 8, *D*, *H*, and *L*, shows the striatal activations of the BGO/DA units in the second last type-1 and type-2 trials within 4,000 trials in the cases where the RNN weights were initialized to excitation-dominant (0.2; Fig. 8*D*) or either of the two manipulations was applied after 3,200 trials (Fig. 8*H*,*L*). Compared with the results without manipulation (Fig. 3*C*) where there appeared activation spanning from a cue to a reward, which means that the credit of the reward was assigned to the preceding states after the cue, no such activation was formed and instead activation following reward receival was formed, indicating that correct credit assignment was failed and spurious credit assignment was formed.

### Fixed cortical state representation and basal-ganglia learning

Thus far we assumed that cortical RNN receives DA so that state representation is trained by TD-RPE. However, many cortical regions receive sparse DA projections, and also there may be conditions when cortical DA actions are not strong. In such cases, cortical state representation may be mainly defined by external (e.g., sensory or motor) inputs and would remain stationary rather than continually updated by TD-RPE. Here we consider such a case. Specifically, we simulated the same task with two trial types by a model with fixed state representation, in which each time-step of each trial type including the ITI was represented by specific activation of a fixed subset of cortical units (without explicitly modeling RNN dynamics) while the cortex → striatum and striatum → BGO/DA weights were updated by TD-RPE (similarly to the original RB model; Millidge et al., 2024). We then examined whether excessive cortical activity induces striatum ↔ DA anti-alignment in this model too, as in the full OVRNN-RB, by varying the level of common baseline persistent activity of all the cortical units.

**Figure 9.**
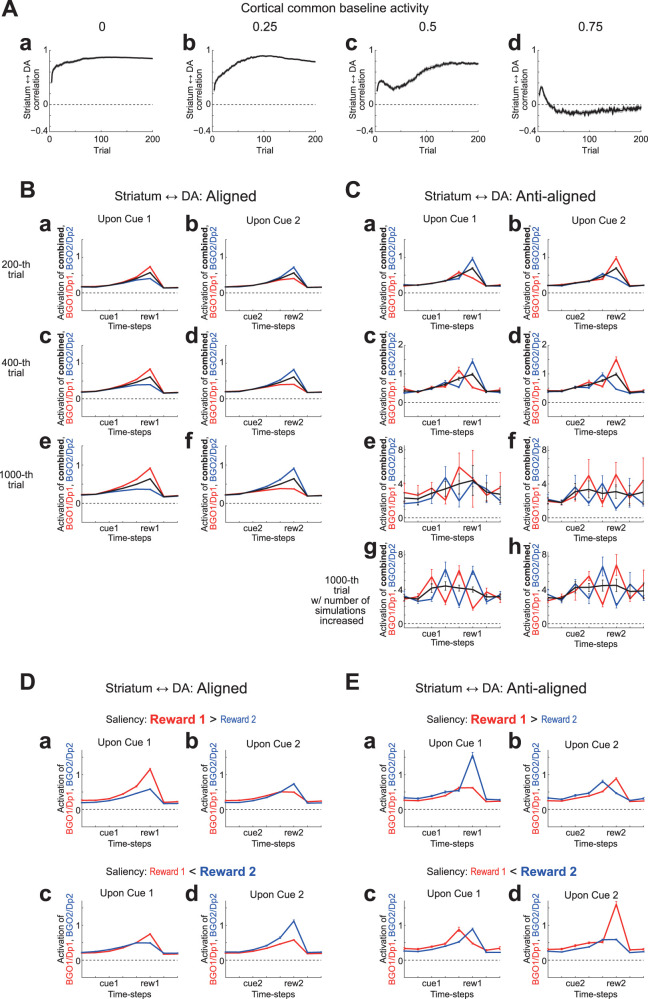
Results with fixed state representation. ***A***, Cortical persistent activity-induced striatum ↔ DA anti-alignment in a model with fixed state representation, in which each time-step of each trial type in the task was represented by specific activation of a fixed subset of cortical units. Panels ***a–d*** show the across-trial evolution of the correlation coefficient between the striatum-DA weights and DA-striatum weights (*r*_SD&DS_) in the cases where all the cortical units have common baseline persistent activity of 0 (***a***), 0.25 (***b***), 0.5 (***c***), or 0.75 (***d***), respectively. ***B***, ***C***, Learning of state values in the model with fixed state representation with striatum ↔ DA alignment (***B***) or anti-alignment (***C***). Striatal activations of the DA units (red, Dp1; blue, Dp2) and their mean (black line, indicating the combined state values) in the last type-1 trial (***a***, ***c***, ***e***, ***g***) and type-2 trial (***b***, ***d***, ***f***, ***h***) within 200 (***a***,***b***), 400 (***c***, ***d***), or 1,000 (***e–h***) trials [error bars indicate ± SEM across 100 (***a–f***) or 1,000 (***g***, ***h***) simulations]. ***D***, ***E***, Reward-specific modulations of striatal activations of BGO/DA units by saliency in the case with striatum ↔ DA alignment (***D***) and their impairments in the case with anti-alignment (***E***). ***a***, ***b***, Striatal activations of BGO1/Dp1 (red) and BGO2/Dp2 (blue) units in type-1 (***a***) and type-2 (***b***) trials in the condition where reward 1 was motivationally highly salient whereas reward 2 had a low salience. ***c***, ***d***, Results for the condition where rewards 1 and 2 had low and high motivational saliences, respectively.

When there was no baseline persistent activity, i.e., only a subset of cortical units corresponding to each time-step of each trial type were active and the other units had no activity (Fig. 9*Aa*), the correlation between the striatum → DA weights and the (randomly fixed) DA → striatum weights rapidly increased and remained positive, meaning a rapid stable formation of alignment. Even in the presence of low common baseline (persistent) activity of cortical units (Fig. 9*Ab*), the striatum ↔ DA correlation smoothly increased and reached a positive steady-state value. A further increase in common baseline activity made the alignment (striatum ↔ DA correlation) slower and for short period of time alignment could decrease (Fig. 9*Ac*). In some simulation instances, we did observe anti-alignment occurred. This anti-alignment became more common when we further increased the persistent activity. For strong baseline activity, striatum ↔ DA correlation was weak and negative, on average (Fig. 9*Ad*), indicating absence of alignment or anti-alignment. Thus, excessive cortical activity-induced striatum ↔ DA anti-alignment can be considered to be a general phenomenon that occurs regardless of whether cortical state representation is fixed or trained, although persistent activity might be more likely to be formed in prefrontal regions with rich recurrent excitation.

Next, we examined whether state values could be learned even under striatum ↔ DA anti-alignment if proper state representation is given and fixed. To do so, we examined two variants of the model with fixed state representation, in which the striatum → DA weights were fixed and were manually either aligned or anti-aligned to the fixed random DA → striatum weights, i.e., **W**_SD_ was set to be a transpose of **D**_S_ (aligned case) or **D**_S_ with two columns swapped (anti-aligned case) while the cortex → striatum weights were kept learnable.

With striatum ↔ DA alignment, striatal activations acquired state values within 200 trials (Fig. 9*Ba*,*b*). Striatal activation of BGO1/Dp1 units, which was activated by reward 1, was stronger in type-1 trials (with cue 1 and reward 1; Fig. 9*Ba*) whereas striatal activation of BGO2/Dp2 units, activated by reward 2, was stronger in type-2 trials (Fig. 9*Bb*), as in the case of the full OVRNN-RB (Fig. 3*C*). Calculating the combined (i.e., reward identity-irrespective) state values by taking an average of the striatal activations of BGO1/DA1 units and BGO2/DA2 units, they were similar across the two trial types (Fig. 9*Ba*,*b*, black lines).

In contrast, in the model with striatum ↔ DA anti-alignment (Fig. 9*Ca*,*b*), striatal activations of BGO1/Dp1 and BGO2/Dp2 units were stronger in type-2 and type-1 trials, respectively. However, formation of these activations following the cue appears still better than the cases of the full OVRNN-RB with aberrant persistent activity (Fig. 8*D*,*H*,*L*), where striatal activation occurred following the reward rather than the cue. Also, the combined state values (i.e., mean activations of BGO1/Dp1 and BGO2/Dp2) look similar to those in the model with striatum ↔ DA alignment. Thus, it could be said that striatum ↔ DA anti-alignment allows learning of state values (i.e., credit assignment of reward) to a certain degree if proper state representation is provided. Notably, however, when more trials were experienced, the pattern of activations became unstable in the model with striatum ↔ DA anti-alignment but not in the model with alignment (Fig. 9*Bc–f*,*Cc–h*).

Striatum ↔ DA anti-alignment is expected to impair reward-specific motivational control, as explained before in a schematic way (Fig. 6). Here we examined it in more detail using the model used just above with fixed state representation and the striatum → DA weights fixed to be either aligned or anti-aligned to the DA → striatum weights. In the model with striatum ↔ DA alignment, when motivational salience of reward 1 was high and that of reward 2 was low (e.g., food and drink when hungry), striatal activation of BGO1/Dp1 units upon cue 1 was particularly enhanced (Fig. 9*Da*,*b*), indicating that expectation of the salient reward 1 upon cue 1 was specifically boosted. Conversely, when motivational salience of reward 1 was low and that of reward 2 was high (e.g., food and drink when thirsty), striatal activation of BGO2/Dp2 units upon cue 2 was particularly enhanced (Fig. 9*Dc*,*d*). Thus, the model with striatum ↔ DA alignment achieved proper reward-specific motivational control.

In contrast, in the model with striatum ↔ DA anti-alignment, when motivational salience of reward 1 was high and that of reward 2 was low, striatal activation of BGO2/Dp2 (rather than BGO1/Dp1) units upon cue 1 was particularly enhanced (Fig. 9*Ea*,*b*). Conversely, when motivational salience of reward 1 was low and that of reward 2 was high, striatal activation of BGO1/Dp1 (rather than BGO2/Dp2) units upon cue 2 was particularly enhanced (Fig. 9*Ec*,*d*). These results indicate that striatum ↔ DA anti-alignment impairs reward-specific motivational control, failing to boost the expectation of salient reward while boosting that of irrelevant reward.

### Simulated inputs to the striatum and possible correspondences to experimental results

The motivational impairments caused by striatum ↔ DA anti-alignment (Figs. 6*C*,*D*, 9*E*), which could be induced by cortical excessive excitation in the model, are reminiscent of the negative symptoms of schizophrenia such as anhedonia, avolition, or irrelevant motivation. Meanwhile, the failure in credit assignment (Fig. 8*D*,*H*,*L*), which could also be induced by cortical excessive excitation in the model, are reminiscent of the positive symptoms of schizophrenia such as delusion or hallucination. Given these together with suggestions that cortical excessive excitation is a major cause of schizophrenia (Lewis et al., 2005; Insel, 2010; Grace, 2016; Howes and Shatalina, 2022), we examined how the OVRNN-RB model predicts cortical excessive excitation-induced changes in the inputs to, and neurotransmitters in, the striatum and compared them to reported experimental results in schizophrenia patients (SZ) versus healthy-control participants (HC).

**Figure 10.**
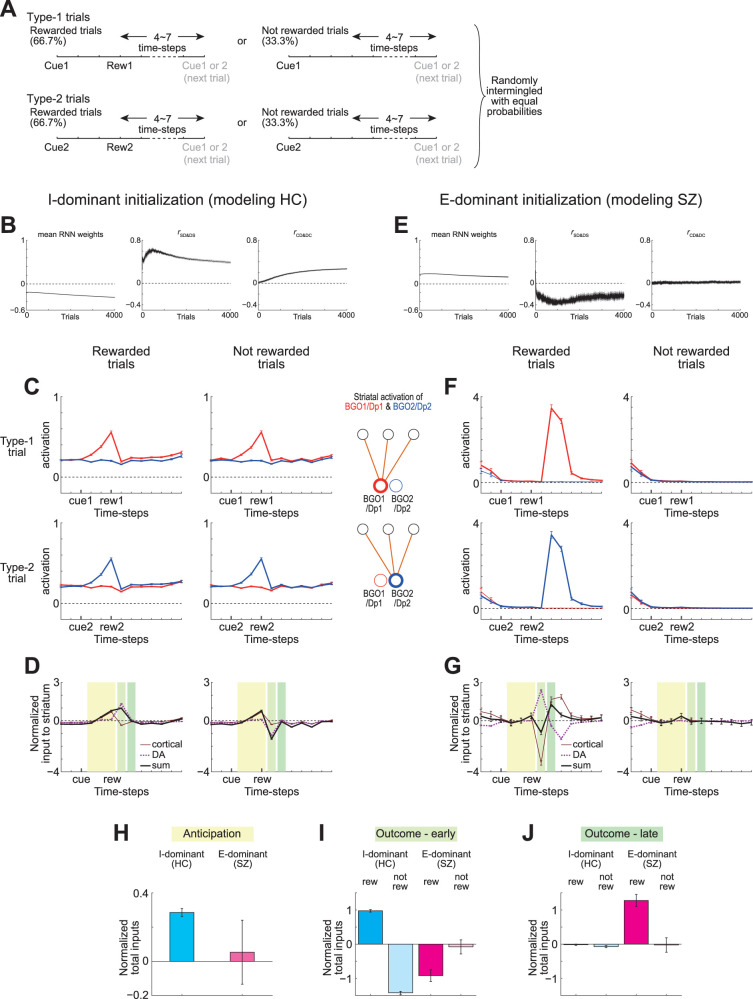
Simulated inputs to the striatum in a probabilistic version of the task. ***A***, The probabilistic version of the task, in which reward was obtained in two-thirds (i.e., 66.7%) of trials. ***B–D***, Results for the OVRNN-RB model with the RNN weights initialized to inhibition (I)-dominant [−0.2, modeling healthy-control participants (HC)]. ***B***, The E/I balance (mean RNN weights, left panel), correlation coefficient between the striatum-DA and DA-striatum weights (middle panel), and correlation coefficient between the cortex (RNN)-striatum-DA connections and the DA-cortex (RNN) weights (right panel). ***C***, Striatal activations of the BGO/DA units (red: BGO1/Dp1, blue: BGO2/Dp2) in the second last type-1 rewarded or not-rewarded trial (top) and type-2 rewarded or not-rewarded trial (bottom) within 4,000 trials. ***D***, Cortical (brown line) and DA (purple dotted line) inputs to the striatum, from each of which the average across the last 100 trials was subtracted, and their sum (i.e., total inputs to the striatum; black line) in the second last rewarded trial (left panel) or not-rewarded trial (right panel) within 4,000 trials (error bars indicate ±SEM across 100 simulations); results for the two trial types (i.e., cue/reward types) were averaged. ***E–G***, Results for the model with the RNN weights initialized to excitation (E)-dominant [0.2, modeling schizophrenia patients (SZ)]. ***H–J***, Total inputs to the striatum for the reward anticipation phase (average over post-cue to reward time-steps, ***H***), outcome-early phase (time-step just after reward, ***I***), and outcome-late phase (two time-steps from reward, ***J***) in the rewarded and not-rewarded trials in the I-dominant/HC and E-dominant/SZ cases.

Neuroimaging studies have shown that SZ and HC show differential activations of brain regions including the striatum upon reward anticipation or outcome feedback, as well as good versus bad outcome in probabilistic task (Juckel et al., 2006; Schlagenhauf et al., 2009; Radua et al., 2015; Leroy et al., 2020; Zeng et al., 2022). In order to examine the behavior of the OVRNN-RB model in reference to those results, we conducted simulations of a probabilistic version of the task, where reward was probabilistically obtained in two-thirds (i.e., 66.7%) of trials (Fig. 10*A*), with OVRNN-RB whose RNN weights were initialized to either inhibition(I)-dominant (modeling HC) or excitation(E)-dominant (modeling SZ).

In the I-dominant (HC) case, both cortex ↔ DA and striatum ↔ DA alignments (Fig. 10*B*), as well as reward-specific activations of BGO/DA units (i.e., reward-specific value anticipation; Fig. 10*C*), were achieved. This is similar to the results of the original nonprobabilistic task. In contrast, the E-dominant (SZ) model led to anti-alignment of striatum ↔ DA and no alignment of cortex ↔ DA (Fig. 10*E*) and also impairment of reward-specific value anticipation and aberrant BGO/DA activation following reward (Fig. 10*F*). Again this is similar to the results of the nonprobabilistic version of the task. Notably, in the E-dominant (SZ) case, there was little activation of BGO/DA in not-rewarded trials (Fig. 10*F*, right).

How the activities in the model relate to fMRI BOLD signals is unclear. Nonetheless, there are suggestions that fMRI BOLD signals predominantly reflect the input to the region (Logothetis et al., 2001) and striatal BOLD signals are correlated with DA release (Schott et al., 2008) and RPE (Rutledge et al., 2010), which is presumably encoded by DA (Montague et al., 1996; Schultz et al., 1997). With these in mind, we calculated the inputs from the cortical RNN units and the DA units to all the striatal units (with the corticostriatal and DA-striatal weights multiplied). Results for the two trial types (i.e., cue/reward types) were averaged, while rewarded and not-rewarded trials were merged for the analysis of the reward anticipation phase but separated for the outcome phase. We looked at the cortical and DA inputs and their sum at the second-last trial within 4,000 trials, from which the average across the last 100 trials was subtracted, considering that slow drifts or changes of BOLD signal are typically filtered out in fMRI analyses.

In the I-dominant/HC case (Fig. 10*D*), the corticostriatal input ramped from cue to reward, reflecting the learned (temporally discounted) state value, while the DA input showed positive and negative phasic responses after the reward timing in the rewarded and not-reward trials, respectively, reflecting TD-RPE. In contrast, in the E-dominant/SZ case (Fig. 10*G*), the cortical input hardly ramped from cue to reward, reflecting the impaired state value formation, while showing a large positive phasic response after reward, which is considered to be related to the aberrant striatal activation at this timing (Fig. 10*F*, left). The DA input showed a larger positive phasic response after reward and did not show a negative response after no reward, reflecting the impaired formation of state value and thus of TD-RPE. There were two additional notable points in the E-dominant/SZ case. First, the positive response of the DA input after reward was followed by a negative response, which is considered to be related to the aberrant striatal activation (Fig. 10*F*, left). Second, the cortical input showed a prominent drop after reward. This is considered to be because the weights from the reward sensation-encoding (sensory cortical) observation unit to the RNN were learned to become negative through the negative DA/TD-RPE.

Given these patterns of cortical and DA inputs, their sum, i.e., the total inputs to the striatum, exhibited differential patterns between the I-dominant/HC and E-dominant/SZ cases as well as between the rewarded and not-rewarded trials (Fig. 10*D*,*G*, black lines). During the reward anticipation phase (i.e., average over post-cue to reward time-steps; Fig. 10*D*,*G*,*H*, yellow shading), there was a larger total inputs in the I-dominant/HC case than in the E-dominant/SZ case in both rewarded and not-rewarded trials. At the outcome-early phase (i.e., time-step just after reward; Fig. 10*D*,*G*,*I*, yellow-green shading), there were positive and negative total inputs in the rewarded and not-rewarded trials, respectively, in the I-dominant/HC case, whereas there were negative and near-zero total inputs in the rewarded and not-rewarded trials, respectively, in the E-dominant/SZ case. Then, at the outcome-late phase (two time-steps from reward; Fig. 10*D*,*G*,*J*, green shading), positive total inputs were observed in rewarded trials in the E-dominant/SZ case whereas near-zero total inputs were observed in the other conditions.

If the total inputs could be considered as a proxy of fMRI BOLD signals, these patterns are largely in line with, and could explain the heterogeneity in, the experimental results. Regarding the reward anticipation phase, meta-analyses (Radua et al., 2015; Leroy et al., 2020; Zeng et al., 2022) have shown hypoactivation of striatum in SZ compared with HC, and the recent analysis (Zeng et al., 2022) also showed its association with negative symptoms. The smaller total inputs to the striatum in our E-dominant/SZ simulation than in the I-dominant/HC simulation [Fig. 10*H*; Wilcoxon rank sum test (same below) *p* = 2.7 × 10^−15^] are consistent with this. As for the outcome feedback phase, previous results (Radua et al., 2015; Zeng et al., 2022) look more heterogeneous. Although a recent meta-analysis focusing on whole-brain SZ-HC comparison (Zeng et al., 2022) found hyper-activation of striatum in SZ with no particular sensitivity to a single study or subgroup difference, only three of ten included studies obtained this direction of result while one obtained the opposite direction, and regarding five nonincluded region-of-interest-based studies, two obtained the same direction while other two obtained the opposite direction [Zeng et al. (2022), their Table S1]. This is in contrast to the results for the reward anticipation phase, for which none of included and nonincluded studies obtained the opposite direction. In our model, at the outcome-early phase of rewarded trials (Fig. 10*I*), the E-dominant/SZ case exhibited smaller total inputs to the striatum than the I-dominant/HC case (*p* < 1.0 × 10^−16^) because the drop of cortical input was more prominent than the increase of DA/TD-RPE. However, if the contributions of the cortical and DA inputs to the total inputs, which were arbitrarily set to 1:1, were set differently (i.e., weighing DA more), the E-dominant/SZ case could exhibit larger total inputs. Also, at the outcome-late phase of rewarded trials (Fig. 10*J*), the E-dominant/SZ case exhibited larger total inputs than the I-dominant/HC case (*p* = 3.0 × 10^−16^). These results could potentially explain the heterogeneity in the experimental results. Besides, although we refrained from considering much about cortical BOLD signal because our model does not describe major inputs such as those from thalamus, the drop of cortical activity upon reward reception in the E-dominant/SZ case could potentially explain hypoactivation of mPFC/DLPFC in SZ at the outcome phase shown in the meta-analysis (Zeng et al., 2022).

Looking at the difference between rewarded and not-rewarded trials, larger total inputs at the outcome-early phase in the rewarded trials were observed in the I-dominant/HC model whereas the E-dominant/SZ model exhibited the opposite pattern (Fig. 10*I*). These differential responses to good and bad outcomes in the I-dominant/HC and E-dominant/SZ models look similar to experimentally observed responses of ventral striatum to success and no success in HC and SZ, albeit in loss-avoidance rather than reward trials [Schlagenhauf et al. (2009), their Fig. 3B].

So far we examined the cortical and DA inputs in the model, from which we subtracted the averages across the last 100 trials as mentioned above. Looking at these averages themselves, there were prominent differences between the I-dominant/HC and E-dominant/SZ cases. Specifically, the average cortical and DA inputs were 1.1 ± 0.4 (SD across simulations) and −0.046 ± 0.014 in the I-dominant/HC case and 28.6 ± 9.9 and −0.005 ± 0.012 in the E-dominant/SZ case. As such, there were much larger baseline cortical input (*p* = 2.7 × 10^−34^) and also larger (less negative) baseline DA input (*p* = 2.2 × 10^−30^) in the E-dominant/SZ case than in the I-dominant/HC case. These differences are in line with the suggested increases of glutamate and DA in the striatum in SZ (reviewed in Howes et al., 2024).

### Possible changes in the degree of plasticity

In our model as well as in the RB model (Millidge et al., 2024), the striatum → BGO/DA weights constitute a basis of the preferences for various rewards, and their differences across individuals define the individual differences in the reward preference. So far, we assumed that the striatum → BGO/DA weights are always fully plastic. However, reward preference in actual animals/humans would not be always plastic. Rather, reward preference may be flexibly acquired in an early phase of development [i.e., critical period (CP); Yang et al., 2012] but may then become largely stabilized, potentially through developmental changes in the degree of plasticity of BGO/DA synapses. This possibility appears to be supported by a recent finding (Karube et al., 2025) that the perineuronal net surrounding parvalbumin (PV)-expressing neurons, which has been shown to regulate CP in the cortex (Pizzorusso et al., 2002; Hensch, 2005), is formed also in BGO [the entopeduncular nucleus and substantia nigra pars reticulata (SNr)] and shows an increase across development. Given this, it is conceivable that in certain psychiatric disorders including schizophrenia, the striatum → BGO/DA weights become highly plastic again, i.e., its CP reopens, presumably in response to cortical E/I imbalance and/or other events, including microglial activation, which regulates cortical CP (Do et al., 2015; Vinogradov et al., 2023) and was shown in the midbrain/SN of subgroups of schizophrenia patients and model mice (Purves-Tyson et al., 2021; Rodrigues-Neves et al., 2022).

**Figure 11.**
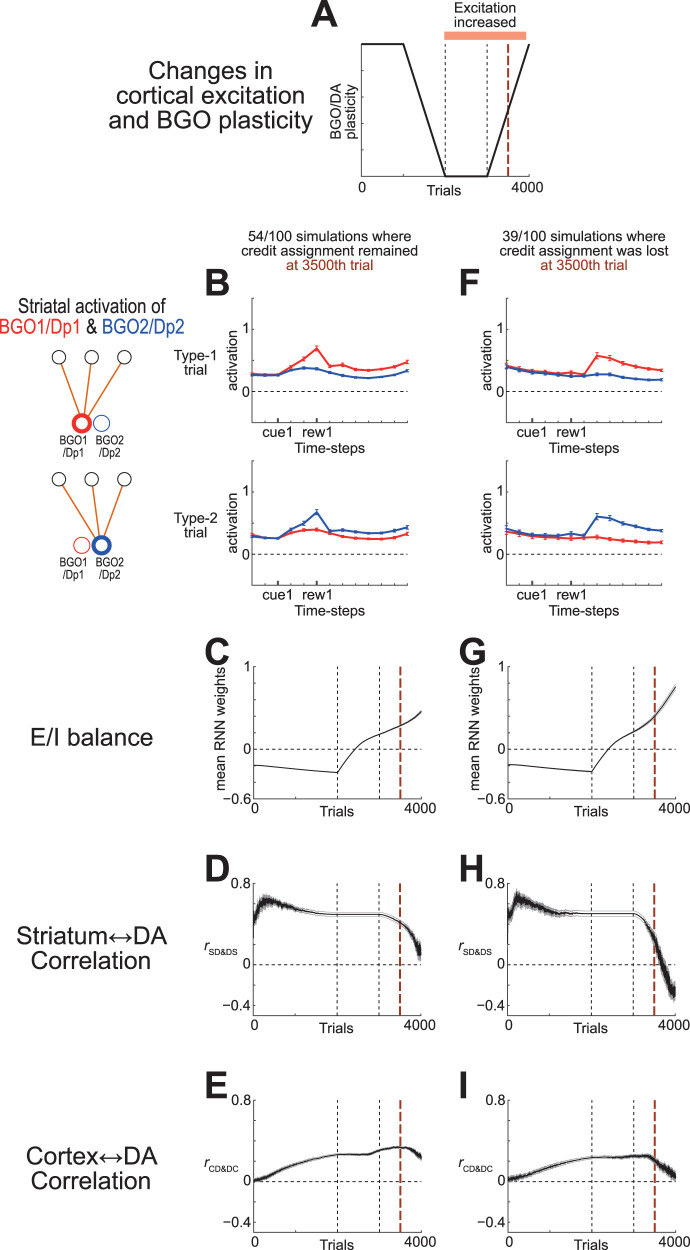
Simulations incorporating changes in the degree of plasticity. ***A***, Assumed changes in the degree of plasticity. The learning rate for the striatum → BGO/DA weights (black line) was initially high (the same rate as in the previous simulations) and decreased to 0 [1,001th–2,000th trials, simulating the closure of critical period (CP)] and then re-increased to the original rate (3,001th–4,000th trials, simulating the hypothesized reopening of CP in schizophrenia). The top orange bar indicates that after 2,000 trials, a small positive value (0.0001) was added to each RNN weight at each time-step (modeling the E/I imbalance). The brown dashed vertical bar indicates 3,500th trial. ***B–E***, Striatal activations of the BGO/DA units in the second last type-1 trial (***B***, top) and type-2 trial (***B***, bottom) within 3,500 trials, cortical E/I balance (***C***), striatum ↔ DA correlation (***D***), and cortex ↔ DA correlation (***E***) in 54 out of 100 simulations, in which the mean activation of the two BGO/DA units averaged across the two trial types at the reward timing was larger than the values at two time-steps earlier and two time-steps later (meaning that proper credit assignment of reward still largely remained). ***F–I***, Results for 39 out of the remaining 46 simulations, in which the mean activation of the two BGO/DA units averaged across the two trial types at the reward timing was smaller than the value at two time-steps later (meaning that proper credit assignment of reward was largely lost and spurious credit assignment was made).

Therefore, we examined how incorporating changes in the degree of plasticity, together with the change in the E/I balance, affected the performance of OVRNN-RB. Although it is impossible to precisely simulate the developmental changes in the degree of plasticity, which should occur over much longer time scales than the time scales in our model, we assumed that plasticity of the striatum → BGO/DA weights was initially active but then became inactive, simulating the stabilization of reward preference (Fig. 11*A*). Then, positive shift of the RNN weights, at a milder pace than the simulation in Figure 8, was assumed to begin (at the 2,000th trial), and after a while (at the 3,000th trial), striatum → BGO/DA plasticity was assumed to become reopen. At the 3,500th trial, in 54 out of 100 simulations, proper cue/reward-specific value predictions (i.e., credit assignment) largely remained (Fig. 11*B*), while in 39 out of the remaining 46 simulations, proper value predictions were almost lost (Fig. 11*F*). The correlation between the striatum-DA forward and feedback weights started to decline at the onset of the plasticity reopening (Fig. 11*D*,*H*), with a more accelerated pace in the simulations with early degradation of value predictions (Fig. 11*H*). Notably, degradation of state representation/value (i.e., credit assignment) could occur before the striatum ↔ DA alignment became totally reversed (at 3,500th trial; Fig. 11*F*) while a mild degradation of the alignment preceded it, and there was a large variety across simulations. These results appear to be potentially in line with the temporal progression of schizophrenia, which is typically diagnosed by a prominent episode of positive symptoms (psychosis) while relatively mild negative symptoms often precede it (Correll and Schooler, 2020).

OVRNN-RB with the changes in the degree of plasticity could potentially further explain why negative symptoms persist after medication that improves positive symptoms (Correll and Schooler, 2020; Marder and Umbricht, 2023). Specifically, if medication shifts/restores the E/I balance back toward inhibition (possibly through blocking cortical D2 receptors, whose activation could potentially shift the balance toward excitation; cf. Seamans et al., 2001; Curtin et al., 2023, 2024; Lányi et al., 2024) but simultaneously closes the hypothesized abnormally heightened striatum → BGO/DA plasticity, the striatum ↔ DA forward and feedback connections would become fixed before alignment is (fully) recovered. Then, as exemplified in the results shown in Figure 3*M–P* where the striatum → DA connections were fixed to be random, reward-specific activation of BGO/DA (motivational control) cannot be achieved while reward-nonspecific value anticipation upon cue (credit assignment) can still be formed albeit to a limited degree (but without spurious credit assignment as seen in Fig. 8*D*,*H*,*L*). This could potentially correspond to that negative symptoms (motivational impairments) persist while positive symptoms (impaired credit assignment) are improved at least to a certain degree.

### Prediction of the OVRNN-RB model: effects of temporal discounting

**Figure 12.**
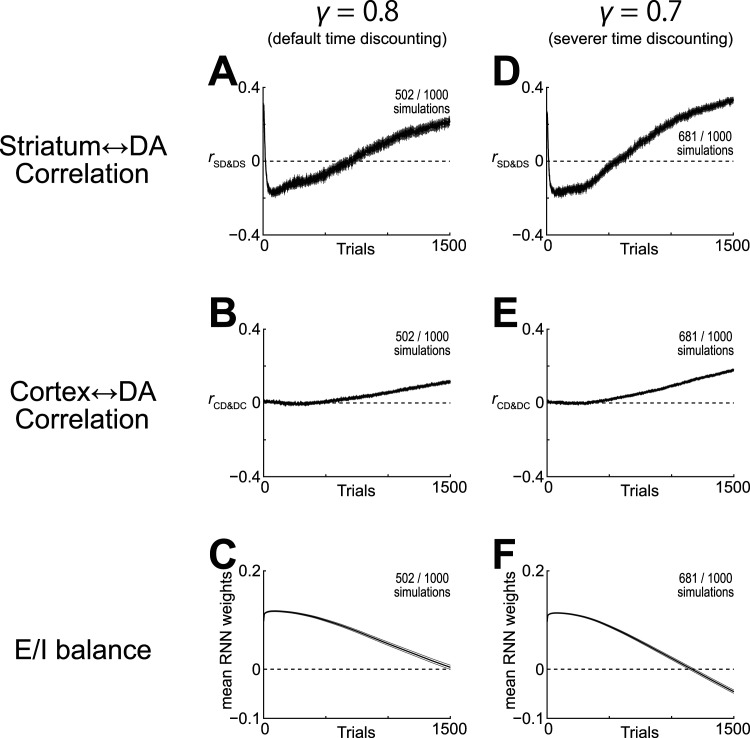
Effects of temporal discounting on the dynamics of OVRNN-RB with two DA units, with the E/I balance initialized to be excitation-dominant (mean RNN weight = 0.1). ***A–C***, Results with the default value of time discount factor (*γ* = 0.8). ***D–F***, Results with a smaller time discount factor (*γ* = 0.7; i.e., severer temporal discounting).

Here we present a prediction of OVRNN-RB through simulations. The key suggestion of this model is that cortical persistent activity due to excessive excitation degrades/reverses the alignment of striatum-DA connections, resulting in functional impairments. Because update of the RNN weights was assumed to depend on DA-encoding TD-RPE: **C**_RD_***r***(*t*) + *γ***W**_SD_***y***(*t* + 1) − **W**_SD_***y***(*t*) (where *γ* is the time discount factor; see the Materials and Methods for details), if *γ* is smaller, i.e., temporal discounting is severer, TD-RPE, and thereby the RNN weight update would tend to be more negative, presumably preventing persistent activity. Indeed, when *γ* was changed from the original 0.8 to 0.7 in the simulation with the mean RNN weight initialized to 0.1, simulation instances with a learning failure after the initial 100 trials became less frequent (498 to 319 out of 1,000), and in the remaining simulation instances, negative correlation (anti-alignment) between the striatum-DA and DA-striatum weights turned into positive at earlier timings (Fig. 12, compare *A*, *D*). As such, our model predicts that the degree (severity) of temporal discounting is negatively associated with cortical excessive excitation and functional impairments. Notably, while temporal discounting is known to be positively associated with several other psychiatric disorders, results for schizophrenia were mixed (Keidel et al., 2025) with either positive (Heerey et al., 2007) or negative (L. Wang et al., 2018) association reported. The latter result, though potentially due to technical issues discussed in that study, is in line with our prediction, and further tests, including those using shorter delays (on the order of seconds) and examining/manipulating the E/I balance in animal models, are desired to be executed.

### Feedback alignments in OVRNN-RB: indices of alignment

As an index of alignment of feedforward connections ***f*** and feedback connections ***b***, we used the correlation coefficient *r* = corrcoef(***f***, ***b***), which was adopted in the study of the ancestor Reward Bases (RB) model (Millidge et al., 2024). Meanwhile, the cosine similarity *s* = ***f***·***b***/(||***f***|| ||***b***||) and the angle based on it *θ* = arccos(*s*) have often been used in the feedback alignment literature (the correlation coefficient *r* represents the cosine similarity between the deviations of ***f*** and ***b*** from their means). Therefore, we examined how the angle *θ* developed in OVRNN-RB with the RNN weights initialized to inhibition-dominant (−0.2, as in Fig. 3*A–D*) or excitation-dominant (0.2, as in Fig. 8*A–D*).

**Figure 13.**
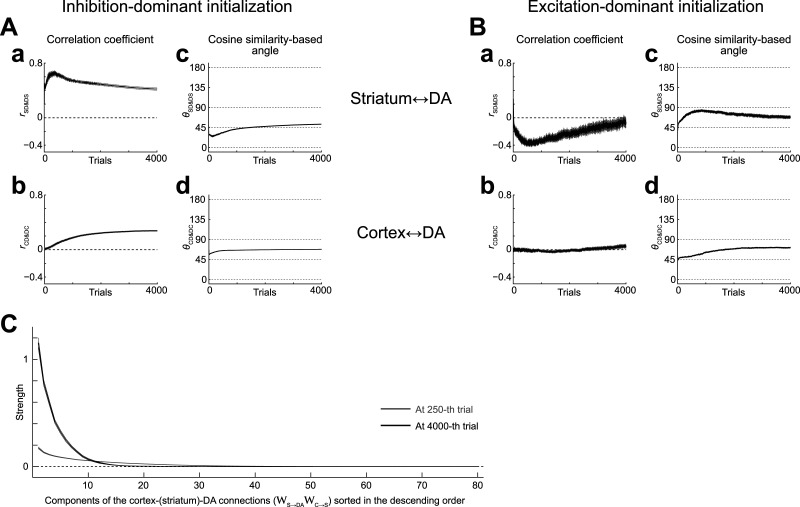
Correlation coefficient and cosine similarity-based angle. ***A***, Across-trial evolution of the correlation coefficient *r* (***a***, ***b***) or cosine similarity-based angle *θ* (***c***, ***d***) between the striatum-DA weights and DA-striatum weights [*r*_SD&DS_ (***a***) or *θ*_SD&DS_ (***c***)] or between the cortex (RNN)-striatum-DA connections (product of the cortex-striatum weights and the striatum-DA weights) and the DA-cortex (RNN) weights [*r*_CD&DC_ (***b***) or *θ*_CD&DC_ (***d***)] in OVRNN-RB with the RNN weights initialized to −0.2. Panels ***a***, ***b*** show the same graphs as those shown in Figure 3*A*,*B*. ***B***, Results in the case with the RNN weights initialized to 0.2. Panels ***a***, ***b*** show the same graphs as those shown in Figure 8*B*,*C*. ***C***, Components of the cortex-(striatum)-DA connections (**W**_SD_**W**_CS_) sorted in the descending order at 250th trial (gray line) and 4,000th trial (black line) in the case with the RNN weights initialized to −0.2. ±SEM (across 100 simulations) are indicated by thin lines but they are almost invisible because SEM was rather small.

The time evolutions of the angle between the striatum → DA and DA → striatum connections (Fig. 13*Ac*,*Bc*) were consistent with the patterns observed for the correlation coefficient (Fig. 13*Aa*,*Ba*). However, while the correlation coefficient drastically differed between the two cases, the angle changed within the range of 0–90° in both cases and the difference between the cases was less drastic. The range limitation for the angle is due to the non-negative constraint that we imposed on the weights to make the model more biological plausible. The range limitation ensured that the feedforward and feedback connection vectors were in the same quadrant (“loosely aligned”) from the beginning, and supposedly promoted learning (Tsurumi et al., 2025), but it also made the difference across conditions less drastic [see also Cheon et al. (2025) for alignment at initialization]. For the cortex ↔ DA connections (Fig. 13*Abd*,*Bbd*), the difference in the angle between the two cases was even smaller. Moreover, even in the inhibition-dominant case, the angle did not actually decrease but slightly increased (Fig. 13*Ad*), while the more prominent increase in the correlation coefficient (Fig. 13*Ab*) indicated that “alignment” in terms of this measure still occurred. Related to this, notably, it has been demonstrated for supervised learning in feedforward networks that random feedback can minimize the error while the cosine similarity-based alignment does not occur unless parameters are regularized (Song et al., 2021).

To understand why and how the correlation coefficient and the cosine similarity-based angle behave differently, here we consider examples. Assume that the feedback weights ***b*** are fixed at ***b*** = (7 8 9) and the feedforward weights ***f*** change from ***f***_1_ = (1 1 5) to ***f***_2_ = (5 3 1). Both ***b*** and ***f***_1_ have monotonically nondecreasing components whereas ***f***_2_ has monotonically decreasing components. Thus, if ***b*** and ***f*** represent the DA → striatum and striatum → DA weights, respectively, ***f***_1_ ensures that the striatum unit receiving the strongest projection from a DA unit most activates that DA unit, enabling proper reward-specific motivational control (Figs. 6*A*,*B*, 9*D*). In contrast, ***f***_2_ would impair (reverse) such modulation (Figs. 6*C*,*D*, 9*E*). For the change from ***f***_1_ to ***f***_2_, the correlation coefficient between ***b*** and ***f*** drastically decreases from +0.866 to −1, changing its sign, whereas the cosine similarity-based angle *θ* changes only slightly from 34.0 to 34.4°. Therefore, the correlation coefficient is considered to sensitively reflect the orders of the weights and thereby the success/failure of reward-specific motivational control, and its use in our model as well as in the RB model could be justified.

The reason for the slight increase in the angle for the cortex ↔ DA connections (even) in the inhibition-dominant case (Fig. 13*Ad*) could be understood using the example. Through learning, the forward connections became sparse (i.e., only a small proportion of connections maintained large strengths; Fig. 13*C*). Given this, now assume that ***f*** changes from ***f***_1_ = (1 1 5) to ***f***_3_ = (0 0 7), i.e., becoming sparse. Then, while the correlation coefficient between ***b*** and ***f*** remains unchanged at +0.866, the cosine similarity-based angle *θ* increases from 34.0 to 49.7°.

## Discussion

In order to mechanistically understand the roles of mesocorticostriatal DA in value learning, motivational control, and cognitive functions, we developed a neurocomputational model, combining previous models that explained either reward-specific motivational control or learning of context-dependent state representation but not them together. Our combined model, OVRNN-RB, provides an integrated account of all these functions.

Normal operation of OVRNN-RB is realized through the occurrence of double alignments. Reward-specific motivational control is achieved through the striatum ↔ DA alignment, as in the ancestor RB model (Millidge et al., 2024), while credit assignment is achieved primarily through the cortex ↔ DA alignment, as in the ancestor OVRNN model (Tsurumi et al., 2025). Demonstration of the occurrence of such double alignments in TD-RPE-trained RNN and its downstream is our novel achievement, although feedback alignments at multiple stages in supervised learning of feedforward networks were previously shown (Lillicrap et al., 2016; Nøkland, 2016).

A key factor for the model's proper operation turned out to be the cortical E/I balance. Excessive cortical excitation degrades/reverses the alignments of cortical and striatal downstream connections to the DA feedback weights, impairing motivational control and credit assignment. This has implications for the symptoms and causes of schizophrenia.

### Implications for schizophrenia

In OVRNN-RB, excessive cortical excitation degrades or even reverses the striatum-DA alignment. If it occurs, even though a particular reward should have a high motivational value, the value of state/action leading to that reward may not be enhanced and can even be diminished. This could manifest as a lack of motivation (avolition) or anhedonia, which are major constructs of negative symptoms of schizophrenia (Strauss et al., 2014; Marder and Galderisi, 2017; Correll and Schooler, 2020; Marder and Umbricht, 2023). Conversely, the value of state/action leading to a reward that should not be highly evaluated under the current context could be enhanced. This could also lead to maladaptive behavior, for example, increased consumption of saturated fat observed in unmedicated patients [Borgan et al., 2019; while obesity, frequently seen in schizophrenia patients (Elman et al., 2006), is considered to be much related to medication].

On the other hand, the positive symptoms of schizophrenia, delusion and hallucination, could originate from difficulties in assigning appropriate credits for a particular event to actions/states that caused or lead to the event (cf. Nour et al., 2025). Since OVRNN-RB achieves credit assignment, its impairment could potentially explain the positive symptoms. Notably, what typically matters in delusion or hallucination is credit assignment for action or intention rather than reward. However, given recent finding of distinct DA signals that encode prediction errors of action (Greenstreet et al., 2025) or potential threat or salience (Menegas et al., 2017, 2018; Akiti et al., 2022; Tsutsui-Kimura et al., 2025) instead of RPE, OVRNN-RB with DA units encoding these prediction errors could potentially explain the positive symptoms.

As for the causes of schizophrenia (Owen et al., 2016; Robison et al., 2020; Howes and Shatalina, 2022), DA (Angrist and Gershon, 1970; Kaar et al., 2020), glutamate (Krystal et al., 1994; Jackson et al., 2004), and developmental (Murray and Lewis, 1987; Marenco and Weinberger, 2000; Murray et al., 2017) hypotheses have been put forward, and they have been proposed to be integrated into changes in the E/I balance (Lewis et al., 2005; Insel, 2010; Grace, 2016; Howes and Shatalina, 2022). However, while previous modeling studies revealed how the E/I imbalance impairs cortical functions (Jardri and Denève, 2013; Murray et al., 2014; Jardri et al., 2017; Lanillos et al., 2020; Calvin and Redish, 2021; Lam et al., 2022), its interaction with RPE-based RL in DA-basal ganglia circuits remained to be explored. Usually, functional role of E/I balance is understood from a “bottom-up” viewpoint, i.e., in the way it changes the network dynamics (e.g., oscillations, synchrony) and the gain of neurons. Here we take a complementary “top-down” [task-variable-based; cf., Langdon et al., 2023] perspective on the functional role of E/I balance. We have shown that in our OVRNN-RB, excessive excitation disrupted the functions [formation of state values (task variables)] by creating misalignment of synaptic connectivity crucial for learning. In our model, we varied the mean RNN weights. This kind of E/I balance is referred to as loose global balance (Hennequin et al., 2017). This is of course simplistic as E/I balance could have a more complicated dynamics (Jardri and Denève, 2013; Murray et al., 2014; Jardri et al., 2017; Lanillos et al., 2020; Calvin and Redish, 2021; Lam et al., 2022). Yet, a change in loose global balance could be a simplest description of empirically suggested E/I imbalance in schizophrenia (Howes and Shatalina, 2022).

Previous studies linked schizophrenia to RL impairments (Gold et al., 2008; Kato et al., 2020; Huys et al., 2021; Millard et al., 2022): flexible goal-directed RL was impaired whereas simple RL was spared (Gold et al., 2008; Morris et al., 2018); impaired performance in RL tasks can in fact come from working memory deficits (Heerey et al., 2008; Collins et al., 2014, 2017); representation of value (Gold et al., 2008) or state (Radulescu and Niv, 2019) may be impaired; and the corticostriatal circuits (Shepherd, 2013) may be affected (Juckel et al., 2006; Schlagenhauf et al., 2009; Radua et al., 2015; Leroy et al., 2020; Millard et al., 2022; Zeng et al., 2022). These findings are broadly in line with our model. Specifically, while OVRNN-RB learns adaptive state representation and value, value learning itself is possible to a certain degree even with anti-alignment if state representation is given in simple situations (Fig. 9*C*), potentially explaining the spared performance in simple tasks. Also, the hypothesized cortical aberrant persistent activity would also impair working memory functions. Moreover, OVRNN-RB could potentially explain some of altered brain activations in patients (Fig. 10). Since OVRNN-RB has overcome the major limitations of conventional RL models, its further elaboration, for example, incorporation of the D1-direct and D2-indirect basal ganglia pathways (Kato and Morita, 2025; Lowet et al., 2025), as well as the effects of D1 and D2 receptor activations in the cortex (Seamans et al., 2001), could potentially provide mechanistic accounts for the diverse symptoms and causes of schizophrenia. Complementary to the transdiagnostic dimensional approach (Gillan et al., 2016; Dalgleish et al., 2020), construction of such first-principle models could greatly help understanding of disorders, as exemplified for addiction (Redish, 2004; Redish et al., 2008; Keiflin and Janak, 2015; Kato et al., 2023).

### Predictions

In addition to the effects of temporal discounting described in the Results, OVRNN-RB provides other predictions. In OVRNN-RB, striatum-DA and cortex-DA double alignments occur. The striatum-DA alignment precedes (Fig. 3, compare *A*, *B*), and it might be completed during early developmental stages [and then stabilized as we assumed in our simulations with changes in the degree of plasticity (Fig. 11)]. The cortex-DA alignment occurs gradually, presumably during learning of behavioral tasks. In rodents, this temporal order of the two alignments could be examined. Gradual cortex-DA alignment during task could also be examined as follows: (1) find a local cortical cite that receives strong DA input upon a particular reward over other rewards, (2) find DA neurons that show strong response to that specific reward, and (3) examine if the DA neurons’ response to a stimulation of that cortical cite increases during learning. Whether such an increase is impaired when the cortical cite is manipulated to entail persistent activity, or in schizophrenia model animals, can also be examined. These could be tested in the prefrontal cortex, but also in the hippocampus, where disruption of reward representation in rat model of schizophrenia risk was experimentally suggested (Speers et al., 2022).
